# Where the bugs are: analyzing distributions of bacterial phyla by descriptor keyword search in the nucleotide database

**DOI:** 10.1186/2042-5783-1-7

**Published:** 2011-07-26

**Authors:** Andrea Squartini

**Affiliations:** 1 Dipartimento di Biotecnologie Agrarie, Università di Padova, Viale dell'Università 16, 35020 Legnaro, Padova, Italy

## Abstract

**Background:**

The associations between bacteria and environment underlie their preferential interactions with given physical or chemical conditions. Microbial ecology aims at extracting conserved patterns of occurrence of bacterial taxa in relation to defined habitats and contexts.

**Results:**

In the present report the NCBI nucleotide sequence database is used as dataset to extract information relative to the distribution of each of the 24 phyla of the bacteria superkingdom and of the Archaea. Over two and a half million records are filtered in their cross-association with each of 48 sets of keywords, defined to cover natural or artificial habitats, interactions with plant, animal or human hosts, and physical-chemical conditions. The results are processed showing: (a) how the different descriptors enrich or deplete the proportions at which the phyla occur in the total database; (b) in which order of abundance do the different keywords score for each phylum (preferred habitats or conditions), and to which extent are phyla clustered to few descriptors (specific) or spread across many (cosmopolitan); (c) which keywords individuate the communities ranking highest for diversity and evenness.

**Conclusions:**

A number of cues emerge from the results, contributing to sharpen the picture on the functional systematic diversity of prokaryotes. Suggestions are given for a future automated service dedicated to refining and updating such kind of analyses via public bioinformatic engines.

## Introduction

The distribution of microbial taxa in relation to environmental factors is a theme of central interest in microbiology and has been addressed by different perspectives and means [1-9]. Several studies investigated community structure of bacterial assemblages assessing the proportions of the different taxonomical groups. These surveys span from highly selective or stressful environmental niches [10], to broader interfaces as the soil [11] or the ocean [12]. Other reports dealt with the wealth of biota composing the hosted microbiomes, as is the case of the human-associated microorganisms [13]. In microbial ecology studies, a particular interest is devoted to understanding which factors do primarily shape the structure of communities. In such sense patterns have emerged pointing towards the importance of soil type [14], or of some chemical conditions as salinity [5]. In the presence of a vast and diverse series of literature indications, efforts have been made to extrapolate consistent data linking taxonomy with habitat preference. Programs have been devised to analyze the output of the next-generation sequencers to compare microbial beta-diversity [15]. Among the issues that are central to the problem of prokaryotic diversity on earth are the size of the sequenced libraries [16], the reliability of the estimators used to draw inferences [17,18], and the question whether the methods in use could be congruent with the goal of assessing the actual diversity [19]. Pyrosequencing approaches from soils have put in evidence numbers of different 16S bacterial sequences ranging from 25000 to 50000 at each site [20]. The same study indicated a difference between agricultural and forest soils in that the former were species-rich but phylum-poor and vice versa. Large scale metagenomic projects as the Sargasso Sea expedition evidenced peaks of previously unknown diversity yielding several thousands of putative novel species at each sampling carried out [21],

In recent reports a large number of published studies has been used as dataset to run comparisons to check the association to different habitat types with increasing levels of hierarchy [8,9]. In these reports authors find relatively low numbers of environment-specific bacterial taxa, and indicate that clear-cut specialization does not appear to be a widely used strategy in prokaryotes.

As knowledge grows up thanks to the studies on environmental microbiota that continue to appear at an ever faster pace, the need is felt for a comprehensive method that could exploit the vast but dispersed literature that continues to cumulate on the different facets of the microbial world. Such tool should ideally operate with an efficient, possibly automated search engine principle, that could tap on a global and constantly updated bank of information. A major link between the individual research reports and a common archive can be found in DNA sequences.

The constantly increasing size of public gene databases, gathering published and unpublished records, and the possibility to operate in-silico searches with multiple combinations of keywords, offers nowadays a powerful tool for the mining of meaningful data in microbial ecology. In parallel, efforts from the Genomic Standards Consortium have also been made to standardize annotation data by taking into consideration habitat-related ontologies, as for the case of the EnvO project http://www.environmentontology.org/.

In the present work an example of such analysis is presented, which was carried out in the NCBI Entrez nucleotide online facility, looking at each of the bacterial systematic divisions in their association with 48 purposely chosen keyword combinations, that are meant to cover an array of environmental and physiological descriptors. The 24 phyla of the Bacteria superkingdom were included, and, within the division Proteobacteria, the six classes from alpha- to zeta- were individually analyzed. The Archaea superkingdom, as a whole, was also screened.

## Results

The results are presented in different forms. Additional File 1, Table S1 lists the raw data, i.e., the number of records featuring each bacterial division across each of the different descriptor words. The first row of figures (GenBank) is the reference line as it shows the total occurrences for each of the phyla in the nucleotide database. Each of the subsequent rows reports the number of sequences having in common that organism (at phylum level) plus the descriptor word(s) in their flatfile annotation. The data pertaining to the Archaea superkingdom are also shown in this table. This latter option allows a first appreciation of the different association of Bacteria and Archaea with the descriptive keywords. The column reporting the ratio of bacteria over Archaea shows the differential rates at which they occur. With a GenBank general ratio of 7.42 some of the descriptors underline their prevalence with values that can be higher than those for bacteria (Hydrothermal, Volcanic, Rumen), or with a ratio still close to 1 ("Atmosphere", "Anaerobic", "Sediment"). On the contrary other descriptors that record Archaea absence of sequences ("Endophyte", "Phyllosphere" or their extremely limited presence in the database ("Mouth", "Clinical", "Human" "Insect").

A very large portion of the public database features records reporting the "uncultured" term in their description. Although not every uncultured organism has the word "uncultured" in the definition, these amount to 2143037 which is 1.6-fold higher than the value for bacteria classified at phylum level. While this label does not imply the unculturable nature of an organism but could simply be the chosen strategy of access to its nucleotide sequence by PCR or cloning steps, it is interesting to compare the distribution of the 'uncultured' designation. This also allows to infer some field-related differences in scientific approaches, in part due to technical aspects. A ratio with the sum of bacteria is shown in the last column and it can be seen that the descriptors giving rise to the highest values of unculturables are "Anaerobic", "Faeces", and "Rumen". The search over the unculturable term is hereby meant not as an alternative to the one done by phyla as some of the records also bear phyla description in their organism field. It is nevertheless a cross indication enabling to appreciate the prevailing investigation strategies

To appreciate distribution and preferences of phyla within the superkingdom Bacteria, the numerical values presented in Additional File 1, Table S1 were elaborated yielding Additional File 2, Table S2 in which each datum of Additional File 1, Table S1 is compared with the percent proportions for the bacterial divisions occurring in the entire database. The first column (Genbank) shows the percentage proportions at which the taxa occur in the whole database (unassociated with any keyword). Such proportions can be defined as those occurring in the "global database metacommunity" of deposited sequences, a concept that well represents the γ-diversity of our present knowledge. This column serves as reference for all comparisons and, for each descriptor keyword, the percent increase or decrease on those values is indicated. For each phylum the double rows show two numbers; the upper values are the plus- (numbers in black) or minus- (numbers in red) variations, with respect to the GenBank reference percentage of that phylum. The lower values of each row are the numbers of fold increase or decrease of the reference percentage brought about by such variation. For example the Actinobacteria have 157719 occurrences in the whole nucleotide database (Additional File 1, Table S1) which amounts to 11.78% of the bacteria listed thereby (Additional File 2, Table S2, first column). The total on which this percentage is worked out is the sum of occurrences of all the taxa (i.e. 1338869, shown in the column "SUM" in Additional File 1, Table S1). When the organism "Actinobacteria" is instead searched also in association with the word "soil", the query yields a subset of 20870 sequences; the percentage of Actinobacteria within the total of the different groups in the soil-tagged search (100094) is therefore 20.81%. In other words Actinobacteria are 20.81% of all eubacteria which associate with the descriptor "soil".

Instead of showing this value, Additional File 2, Table S2 reports directly its difference from the general database percentage. Therefore as 'Actinobacteria' had a 11.78% value in the generic unassociated database, in the fraction of it associated with "soil", they are enriched to 20.81%, i.e. there is a net positive increase of +9.03. The increase over the baseline percentage is 1.77-fold (from 11.78% to 20.81%). Both these values are shown in Additional File 2, Table S2. The upper one (percentage net variation) especially allows an appreciation of the trends displayed by the numerically abundant groups, while the second (percentage fold increase) ensures to better notice the variations of minority groups, whose proportional variation is little on the total but can be large for that single group. The table is meant to point out in which context would any phylum be either enriched or depleted compared to its global database metacommunity average. The cell colours highlight the positive and negative trends over different thresholds of intensity for an easier identification of the most remarkable differences. Blank (empty) cells are the cases in which, for that taxon, no records exist in association with that given descriptor. The order in which the keywords are presented in the columns starts with a series corresponding to habitats in the broad-scale environment, with extreme ones further on the right. After those, starting with the "Symbiont" tag, there are a series of terms applying to niches of interactive type with higher organisms as hosts or partners. Proceeding further on the table, there is a series of artificial or man-impacted contexts, to end up with some terms relating to biochemical or physiological significance. These descriptors are not meant to be mutually exclusive as some records may contain more than one of these keywords. The table essentially depicts the percent differences among ranks. The higher the positive values, the more that habitat/descriptor stands out as specific for enriching that particular phylum over the rest of other phyla. For the same reason, the more a phylum scores as specific for a limited number of habitats, the less that phylum can be considered as cosmopolitan.

It is important to underline that, for the descriptor-associated sequences, the percentage of each number of occurrences is compared versus the percentage of all records of that phylum present in the whole database (and not versus a sum of the records resulting from the table). This way the results obtained with a given descriptor are independent from those of the other descriptors. Therefore omitting a descriptor or not having included in the present study other, possibly relevant ones, does not affect the results.

The way data are presented in Additional File 2, Table S2 is useful to show trends of enrichment or depletion over the global metacommunity of the database. However that output is a comparison among ranks and may not render the picture of the absolute habitat preferences possessed by each phylum. Such a different view can be obtained again from the raw data of Additional File 1, Table S1 by a different elaboration putting in evidence the percent in habitats.

For such purpose in Additional File 3, Table S3, the data show how much percent of the total records present in the database, for a given phylum, are individuated by a certain descriptor/habitat. The results are shown in order of decreasing abundance thus presenting the taxonomical phyla as lists in which the descriptor/habitats are. This arrangement shows, at a glance, which are the most frequently recurring habitats for each of the phyla. The calculations are done from the data in Additional File 1, Table S1. For example, as the Actinobacteria records associating with the word soil are 20870 over 157719 (i.e. 13.23%), that equals to say that "soil" has 13,23% of all Actinobacteria sequences present in GenBank and searched with the criteria specified.

In this table the length of each list and its evenness also allows to appreciate the trend towards either specificity or cosmopolitanism associated to the different phyla. Short lists with presences concentrated at high percentages in the top lines imply higher phylum-level specificity, while long lists, in some cases encompassing all the 48 descriptor words, denote a broader cosmopolitan attitude. The latter situation is the case for Actinobacteria, Bacteroidetes, Firmicutes, Betaproteobacteria, Gammaproteobacteria. Adopting a criterion of being linked to at least 90% of the descriptors to qualify for cosmopolitanism [9], in our case 11 taxa over 29 fall in such category (37.9%). It should nevertheless be remarked that the descriptors used here are not of comparable nature as some coincide with true environmental niches, while some embody a chemical concept. For such reason, in order to extract an ecological insight independent from the keyword heterogeneity, a different analysis has been applied and each set of data, individuated by a given descriptor, has been treated as a community in which the taxonomical resolution is set at phylum (or class) level, and different ecological indexes of diversity and evenness were calculated. These included Simpson's Diversity (Inverse Dominance 1/D, or Hill's N2); Shannon-Wiener's Diversity (H'), Simpson's Evenness (E1/D); Shannon-Pielou's Evenness (J'). For definitions and formulae refer to [22]. Results are shown in Additional File 4, Table S4.

**Figure 1:**
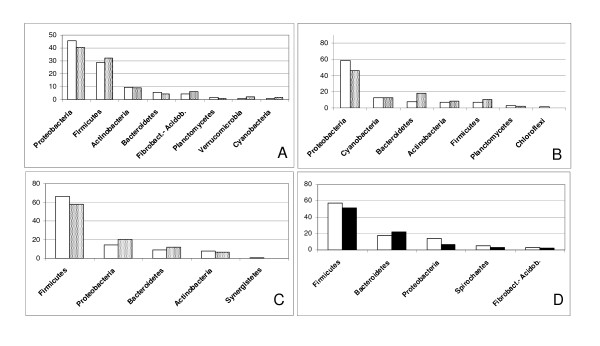
**Verification of the predictive accuracy of the method**. Comparisons between the percentages arising in the present work (white bars) and known community compositions from compiled data of microbial ecology studies (grey and black bars). Cases A,B,C are compared with data drawn from the EnvDB dataset, (options: OTUs, Genbank) A) Terrestrial/Soil/Agricultural; B) Aquatic/Saline water; C) Host associated/Oral; D) Data from 454 sequencing of a single camel rumen.

In order to test the reliability of the in-silico evaluation methodology discussed in the present article, results were compared with available literature data stemming from sequencing projects of actual environmental communities from different habitats. The source used was the EnvDB online compilation [8], featuring a large number of sequences from different studies in classified environments. The results are shown in Figure 1 where the proportions of the phyla found at frequencies higher than 1% are compared with the corresponding virtual values resulting from the present approach. Three representative environments are shown including the agricultural soil, the sea, and the human mouth. The fourth panel is instead a comparison with a more specific habitat subtype, obtained from an analysis of ours targeting the rumen content of the African camel, from which we run a 16S amplicon 454 sequencing yielding over 23000 sequences (Rosselli et al. manuscript in preparation), whose identities were used to compare the community with that generated by the present keyword search using the "Rumen/Ruminal" descriptor. In all these comparisons, it can be observed that the community proportions arising from the database search method orderly agree with those assembled from selected studies, and respect the overall community structures for the different habitats. Such good consistency is verified also notwithstanding the fact that the terms used for the present keyword search are not always coincident with those annotated in the original studies or used in the hierarchical environment classification used at the EnvDB facility. In the specific, the comparisons between the presently generated data and those from known studies used the following pairs of descriptor sets: Agriculture OR crop, vs. Terrestrial/Soil/Agricultural; Seawater OR sea OR marine OR ocean vs. Aquatic/Saline water; Mouth OR oral OR buccal vs.Host associated/Oral.

## Discussion

The synoptic observation of the scenario of bacterial distribution in relation to the search words used (Additional File 2, Table S2) reveals a number of interesting aspects. While some of these are in line with expectations or fall within common microbiological knowledge (supporting nonetheless the trustfulness of the method), many others are less known or offer a novel insightful hint on the preferences and exclusion forces that could drive the prokaryotic associations in natural as well as man-managed environments. Starting to comment from the major groups, the gram-positives Firmicutes are the numerically dominant members of the database (26% of the classified Bacteria), however the table shows how this high average over the other groups is explained and maintained when linked to descriptors of interactivity with warm-blooded hosts and in particular for the nutritional trait, while as regards the majority of environmental type niches, the association shows a depletion over the untagged average values. The Firmicutes taxon is also strongly enriched when linked to words as acid, and resistant.

In the database the second most numerous group is the class Gammaproteobacteria whose abundance is almost as high as the Firmicutes, amounting to 329912 records. Compared to their database mean position they appear depleted in most of the broad environment niches with the exception of seawater, confirmed also by the positive association with the "halophilic" descriptor. They also show a negative trend in the community when searched with the keywords linked to extremophily while being slightly enriched with some of the interactive-type descriptor as "plants", "phyllosphere" or "insects". Their prevalence appears moreover negatively linked with the majority of anaerobic or microaerophilic host-linked environments. The third group, in terms of absolute abundance, are the Actinobacteria. Besides the expected positive links with "Antibiotic" and "Degrading", their proportion in the community denotes a trend of association with non-impacted environments (forest, soil, but not particularly of agricultural type), including dry habitats, with a peak (+32%) in relation to the Atmosphere descriptor, and interesting hints for endophytism. Analyzing together the other classes of the Proteobacteria, the Alpha- confirm their priority place within communities in symbiotic associations with plants, while Betaproteobacteria, besides scoring positive trends for grassland, are definitely highlighted by words as "activated sludge" (+23.45%) and especially by "Oxidizing" (+59.98%). This remarkable surplus is interestingly matched by the complementary word "Reducing" for which an equally high deviation (+60.65%) is displayed by the sister class Deltaproteobacteria, whose frequency is boosted by a 22-fold increment under this descriptor. It appears that the two evolutionarily distinct branches of Beta- and Delta-proteobacteria would have partitioned each towards one of the two main biochemical directions of the redox circle. The latter are correspondingly enriched by the "anaerobic", "aquifer-cave", "sediment" descriptors, and definitely in minority under "human", "clinical", "fecal", and host-type keywords in general. The Epsilonproteobacteria encompass species which are instead known pathogens, but it is worth remarking that, as a phylum, their peak associations arise with the "Hydrothermal" and "Volcanic" search items. The Zetaproteobacteria have very few records in the database, which pinpoint them as a rather specific phylum associated mostly with the terms "Hydrothermal" and "Oxidizing". Going back to other relatively conspicuous phyla, the Bacteroidetes (3.79% of the unassociated metacommunity phyla) score their competitive best when matched with "Feces", "Intestine", "Bovine", "Rumen", but yield 'plus' values also with the "Wetland" and "Lake" descriptors. The photosynthetic Cyanobacteria confirm their aquatic nature but peak even higher at the Desert descriptor, remarking the notion of their main role in communities at the surface crust or at the hypolithic interface of dry ecosystems. A number of other various associations can be observed with the other minor phyla such as the confirm of Acidobacteria as most represented soil-dwelling bacteria; the strong enrichment of Verrucomicrobia with terms as "Pasture" and "Grassland"; the stronger association of Gemmatimonadetes with "Forest", the "Rumen" specificity of Dictyoglomi, the "Mouth" and "Thermophilic" preferences for Synergistetes.

As regards Additional File 3, Table S3 the results enable to view aspects that are different from those shown by the elaboration presented in Additional File 2, Table S2. For example, while in the previous analysis we learned that the percent Cyanobacteria among the other ranks was mostly enhanced by the descriptor "Desert", Here we see that the habitat that nevertheless contains most of their records is associated with the word "Seawater" that alone accounts for 28.14% of their database sequences. Therefore "Desert" preferentially enriches the search result of Cyanobacteria over all other groups, although it may not necessarily be the descriptor that comprises their majority. In the present table we see that in fact the keyword that gathers the highest proportion of Cyanobacteria is, as reported above, "Seawater". Additional File 3, Table S3 therefore allows to see which is the dominant environment/descriptor for each of the phyla and which are respectively the following ones in terms of decreasing abundance. Most phyla show percentage saturation (i.e. the sum of values exceeds 100%) which is due to the fact that the descriptors are not exclusive of each other (e.g. some taxa can share thermophylic and seawater and acid etc.). However as each descriptor is treated independently, their possible overlaps do not affect the interpretations. For some minor taxa, (in particular the Chlamydiae) the coverage obtained by the descriptors appears low. This is due to the presence, for this group, of a relatively large set of sequences of genome-type but deposited without sufficient descriptive terms to achieve their filtration. Removing these by further keyword filtering would bring the dominant habitats percentages of the Chlamydiae up to 74% for the dominant descriptor ("Clinical"). As the Chlamydiae amount to only 1.59% of the GenBank bacterial phyla, this would just minimally affect the percentages of the others. Therefore it was preferred to keep the same search criteria used for all phyla to allow unbiased comparisons of the whole data.

From the ecological analyses (Additional File 4, Table S4) obtained upon treating the data as virtual communities, it can be seen that the keywords raising subcommunities at the top positions of the diversity scale are, (with a good agreement of the two indexes), "Alpine", "Sediment", "Wetland", "Hydrothermal", "Volcanic", and "Lake". As regards the evenness, i.e. the degree of distribution equilibrium in the community structure, the top ranking entries are "Endophyte", "Alpine", "Pasture" and "Lake". At the other end of the scale, keywords that seem to provide less diverse arrays of taxa and the least even community structures appear "Food" and "Mouth". This latter datum is in agreement with the recent report of Tamames and coworkers [9], based on a survey of a number of studies, that indicates a more saturated diversity situation for collector curves drawn for oral bacterial communities.

As regards the validation of the principle proposed in this work, the comparisons run with known environmental results (Figure 1) indicate that the method actually works in practice. This evidence supports the view that notwithstanding its simplicity, and the potentiality of a series of fine tuning improvements of bioinformatical kind, the method offers grounds for an inexpensive and time-saving analytical tool for microbial ecology.

To summarize, the type of novel information that can be extracted by this method can be explicated as follows:

1. It can reveal hitherto undetected associations and preferences between given taxonomical groups and environmental facets, in the form of sites, hosts or physico-chemical conditions

2. It allows the use of data to calculate ecological indices (diversity, evenness community similarity etc.) to trace common patterns, parallels, divergences, and trends for each of the habitats under consideration.

3. For all the data that will stem from new individual studies of microbial ecology, the charts allow to run a comparison to verify whether with the case matches the prevailing association with the corresponding habitat descriptors, or to which extent and for which taxa it does diverge from the mean distribution. Such aspect can also suggest the existence of new yet undefined habitat conditions that can explain microbial community composition variation over a given environmental supertype.

4. It can be customized by the user by defining new keywords as well as using multiple combinations of them in order to extract ever-refined information on microbial taxa distribution.

## Conclusions

The perspective introduced by this work was meant to continue addressing the basic postulate put forward by Baas-Becking [23]. The rankings in Additional File 3, Table S3 are clues to address the extent to which "Everything is (or is not) everywhere", while the variation of percentages shown in Additional File 2, Table S2 are conceived to guide our interpretations towards the trends by which "The environment does (or does not) select" bacterial assemblages.

The search presented here was performed during early 2011. As the GenBank database is constantly updated with new deposited sequences from worldwide origin, the result represents a snapshot of the situation at a given moment that nevertheless cumulates decades of research having contributed to the global picture resulting at that point. As the number of records in the database is in the order of millions, the robustness of data plays in favour of their relatively good stability in time. Nevertheless a periodical refreshment would stabilize data or could reveal shifts proportional to the effort for some yet less analyzed divisions. Refinement of the search criteria could also ensure to lower possible biases inherent to the process. In particular the following critical issues are envisaged as requiring attention, (a) the degree of overrepresentation of some particular species on which more extensive research has been devoted, (b) the accuracy of annotation under which the habitats are reported in the records. (c) the inclusion in the bank of many recent next-gen environmental sequences, which are currently held in separate trace and short read archives and whose growing number will in the future outnumber the corresponding Sanger collections.

In fact the exercise presented hereby is meant to show the prototypic concept for a fast and inexpensive data mining principle. The intention is to suggest the introduction of a dynamic analysis that would need to be both implemented in its search criteria and periodically repeated in time, in order to delineate an ever-refined picture, helping to tune up our knowledge on microbial distributions and associations. Ideally, this kind of search, which could be performed also at species-level, could become a routinely automatized and updated bioinformatical service, run by engines of the national database centre itself.

## Methods

Searches were carried out online at the National Centre for Biotechnology Information Website http://www.ncbi.nlm.nih.gov/ in the nucleotide database. The basic syntax used to build the results tables was the following: ('descriptor keyword' OR 'synonymous descriptor keyword') AND 'Phylum name'[Organism] NOT genome.

The descriptive keywords were defined in order to cover different habitats and contexts of environmental, applied, and physiological relevance. The choice of terms took into account the possible variability of words used in the database records, and multiple query terms were adopted when appropriate. As examples: (endophyte OR endophytic); (halophilic OR salt OR saline); (volcano OR volcanic). For some keywords the occurrence of possible different spellings or adjective forms was considered; e.g. (feces OR faeces OR fecal). In each search such descriptors were matched with each bacterial phylum (or class for the Proteobacteria) and the number of records containing both was reported in Additional File 1, Table S1. Among the premises for such screening is the consideration that a vast majority of sequences present in the database are from environmental studies in which whole bacterial communities have been studied by culture-independent methods. Purposely defined search options were adopted to work on datasets satisfying this criterion. The following basic filters were employed: a) the taxonomical rank sought for was to be contained in the [Organism] field of the record. This ensures not to pick up cases where a bacterial division is quoted elsewhere in the record of a sequence belonging to an organism of different taxonomy. b) the sequences belonging to genome sequencing projects were excluded. This leaves out all those records corresponding to thousands of individual sequences belonging to single bacterial strains.

The syntax routinely used for a search was therefore as in the following example:

(Alkaline OR alkaliphilic) AND Actinobacteria[Organism] NOT genome

Further tips were adopted for the choice of some descriptor terms for which a possible confusion or double meaning was likely. For example "Plants" was chosen instead of "Plant" as the latter word could occur also as "industrial plant". Also for some terms that could occur in the authors affiliation a specification was added. For example when searching the descriptor "Lake" specifications with Boolean operators were added to exclude records deposited from Salt Lake City etc. In general however while a minimal degree of "false positives" is inevitably bound to occur with some of the descriptors, the very large dimension of the sampled population (over 2.5 millions of sequences for bacteria), ensures the overall robustness of the operation. Detailed inspections of the resulting records confirmed the accuracy of the search criteria, which, given the rapid and simple mode of operation, represent a very convenient compromise to achieve a reliable picture mirroring microbial distribution through the different niches and in the various biochemical contexts.

## Competing interests

The author declares that they have no competing interests.

## Authors' information

AS, (PhD in Molecular interaction between bacteria and plants earned in 1987), is a faculty member at the University of Padova, Professor of Microbial Ecology features a 30 years-long research experience in applied microbiology.

## Supplementary Material

Additional file 1 **Table S1. Association of phyla or classes with descriptor keywords**. Number of Genbank records of nucleotide sequences referring to the different phyla and featuring the different descriptor keywords in their flatfile text. For descriptors using more than one term, the complete search words are specified by the notes as follows. ^1^Agriculture OR crop; ^2^Grassland OR prairie; ^3^Alpine OR mountain; ^4^Wetland OR marsh OR wetlands OR marshes; ^5^Seawater OR sea OR marine OR ocean; ^6^Aquifer OR groundwater OR karst OR cave; ^7^Volcanic OR volcano; ^8^Atmosphere OR atmospheric; ^9^Hydrothermal OR geothermal; ^10^Halophilic OR salt OR saline; ^11^Psychrophilic OR ice OR glacier OR glacial OR arctic OR permafrost; ^12^Symbiont OR symbiotic; ^13^Endophyte OR endophytic; ^14^Rhizosphere OR root OR rhizospheric; ^15^Phyllosphere OR phyllospheric OR leaf OR leaves; ^16^Insect OR larvae OR moth; ^17^Cow OR bovine OR cattle OR calf; ^18^Rumen OR ruminal; ^19^Intestinal OR intestine OR gastrointestinal; ^20^Mouth OR oral OR buccal; ^21^Feces OR faeces OR fecal; ^22^Antibiotic OR antibiotics; ^23^Degrading OR degradation OR degradative; ^24^Polluted OR pollution; ^25^Activated sludge; ^26^Acid OR acidic OR acidophilic; ^27^Alkaline OR alkaliphilic; ^28^Anaerobic OR anaerobe OR anaerobes. The first row of data shows the total number of occurrences of the nucleotide database featuring each phylum or class name in the [Organism] field, with the exclusion of the genomic projects (NOT genome). The taxonomical groups of Armatimonadetes, Caldiserica, and Lentisphaerae, currently in the process of becoming novel phyla, were at this stage dealt with as candidate phyla awaiting placement. Click here for file

Additional file 2 **Table S2. Distribution differences in ranks**. Elaboration of the data shown in TableS1, expressing the positive (in black) or negative (in red) differences of the percent values with respect to those occurring in the whole database (GenBank column). Upper values: difference over the reference percentage; lower values: fold of increase or decrease of the reference percentage. Click here for file

Additional file 3 **Table S3. Distribution differences in habitats**. Elaboration of the data shown in Table S1, expressing, for each of the phyla, the percent of the total GenBank occurrences associated with each given descriptor, and ordered in decreasing abundance. Click here for file

Additional file 4 **Table S4. Ecological indexing of the descriptor-generated subcommunities. **Elaboration of the data shown in Table S1. Each of the numerical communities individuated by the database filtering with the different descriptors (i.e. each of the rows of Table S1) was treated as a defined ecological assemblage and the following indexes were calculated. Simpson's Inverse Dominance (1/D, Hill's N2); Shannon-Wiener's Diversity (H'), Simpson's Evenness (E1/D); Shannon-Pielou's Evenness (J'). Click here for file

## References

[B1] Horner-Devine MC Carney KM Bohannan BJ An ecological perspective on bacterial biodiversity Proc Biol Sci 2004 271 113 122 10.1098/rspb.2003.2549 15058386 PMC1691570

[B2] Horner-Devine MC Bohannan BJ Phylogenetic clustering and overdispersion in bacterial communities Ecology 2006 87 7 Suppl S100 108 16922306 10.1890/0012-9658(2006)87[100:pcaoib]2.0.co;2

[B3] Green J Bohannan BJ Spatial scaling of microbial biodiversity Trends Ecol Evol 2006 21 501 507 10.1016/j.tree.2006.06.012 16815589

[B4] Martiny JB Bohannan BJ Brown JH Colwell RK Fuhrman JA Green JL Horner-Devine MC Kane M Krumins JA Kuske CR Morin PJ Naeem S Øvreås L Reysenbach A-L Smith VH Staley JT Microbial biogeography: putting microorganisms on the map Nat Rev Microbiol 2006 4 102 112 10.1038/nrmicro1341 16415926

[B5] Lozupone CA Knight R Global patterns in bacterial diversity Proc Natl Acad Sci USA 2007 104 11436 11440 10.1073/pnas.0611525104 17592124 PMC2040916

[B6] von Mering C Hugenholtz P Raes J Tringe SG Doerks T Jensen LJ Ward N Bork P Quantitative phylogenetic assessment of microbial communities in diverse environments Science 2007 315 1126 1130 10.1126/science.1133420 17272687

[B7] Fierer N Breitbart M Nulton J Salamon P Lozupone C Jones R Robeson M Edwards RA Felts B Rayhawk S Knight R Rohwer F Jackson RB Metagenomics and small-subunit rRNA analyses reveal the genetic diversity of bacteria, archaea, fungi, and viruses in soil Appl Environ Microbiol 2007 73 7059 7066 10.1128/AEM.00358-07 17827313 PMC2074941

[B8] Pignatelli M Moya A Tamames J EnvDB, a database for describing the environmental distribution of prokaryotic taxa Environ Microbiol Reports 2009 1 191 197 10.1111/j.1758-2229.2009.00030.x 23765793

[B9] Tamames J Abellán JJ Pignatelli M Camacho A Moya A Environmental distribution of prokaryotic taxa BMC Microbiol 2010 22 10 85 20307274 10.1186/1471-2180-10-85 PMC2850351

[B10] Demergasso C Casamayor EO Chong G Galleguillos P Escudero L Pedrós-Alió C Distribution of prokaryotic genetic diversity in athalassohaline lakes of the Atacama Desert, Northern Chile FEMS Microbiol Ecol 2004 48 57 69 10.1016/j.femsec.2003.12.013 19712431

[B11] Schloss PD Handelsman J Towards a census of bacteria in soil PLoS Comput Biol 2006 2 7 e92 10.1371/journal.pcbi.0020092 16848637 PMC1513271

[B12] Sogin ML Morrison HG Huber JA Welch DM Huse SM Neal PR Arrieta JM Herndl GJ Microbial diversity in the deep sea and the unexplored "rare biosphere" Proc Natl Acad Sci USA 2006 103 15 20 10.1073/pnas.0509693102 16880384 PMC1524930

[B13] Kuczynski J Costello EK Nemergut DR Zaneveld J Lauber CL Knights D Koren O Fierer N Kelley ST Ley RE Gordon JI Knight R Direct sequencing of the human microbiome readily reveals community differences Genome Biology 2010 11 210 10.1186/gb-2010-11-5-210 20441597 PMC2898070

[B14] Girvan MS Bullimore J Pretty JN Osborn AM Ball AS Soil type is the primary determinant of the composition of the total and active bacterial communities in arable soils Appl Environ Microbiol 2003 69 1800 1809 10.1128/AEM.69.3.1800-1809.2003 12620873 PMC150080

[B15] Hamady M Lozupone C Knight R Fast UniFrac: facilitating high-throughput phylogenetic analyses of microbial communities including analysis of pyrosequencing and PhyloChip data The ISME Journal 2010 4 17 27 10.1038/ismej.2009.97 19710709 PMC2797552

[B16] Kemp PF Aller JY Estimating prokaryotic diversity: when are 16S rDNA libraries large enough? Limnol Oceanogr Methods 2004 2 114 125

[B17] Curtis TP Sloan WT Prokaryotic diversity and its limits: microbial community structure in nature and implications for microbial ecology Current Opinion in Microbiology 2004 7 221 226 10.1016/j.mib.2004.04.010 15196488

[B18] Curtis TP Wallbridge NC Sloan W Butlin R, Bridle J Theory, community assembly, diversity and evolution in the microbial world Speciation and Patterns of Diversity 2008 Cambridge University Press 59 76

[B19] Quince C Curtis TP Sloan WT The rational exploration of microbial diversity ISME Journal 2008 2 997 1006 10.1038/ismej.2008.69 18650928

[B20] Roesch LF Fulthrope RR Riva A Casella G Hadwin AKM Kent AD Daroub SM Camargo FAO Farmerie WG Triplett EW Pyrosequencing enumerates and contrasts soil microbial diversity ISME J 2007 1 283 290 18043639 10.1038/ismej.2007.53 PMC2970868

[B21] Venter JC Remington K Heidelberg JF Halpern AL Rusch D Eisen JA Paulsen IT Nelson KE Nelson W Fouts DE Levy S Knap AH Lomas MW Nealson K White O Peterson JD Hoffman J Parsons R Baden-Tillson H Pfannkock C Rogers Y-H Smith HO Environmental whole genome shotgun sequencing: the Sargasso Sea Science 2004 304 66 74 10.1126/science.1093857 15001713

[B22] Magurran AE Ecological Diversity and Its Measurement 1988 Princeton University Press. Princeton NJ

[B23] Baas-Becking L Geobiologie of Inleiding Tot de Milieukunde 1934 Van Stockkum & Zoon. The Hague

