# Supplementary material for: Where the bugs are: analyzing distributions of bacterial phyla by descriptor keyword search in the nucleotide database
Source: Microb Inform Exp. 2011 Jul 26;1:7. doi: 10.1186/2042-5783-1-7 (PMC3372287; doi:10.1186/2042-5783-1-7)
Supplement: Additional file 1 — Table S1. Association of phyla or classes with descriptor keywords. Number of Genbank records of nucleotide sequences referring to the different phyla and featuring the different descriptor keywords in their flatfile text. For descriptors using more than one term, the complete search words are specified by the notes as follows. 1Agriculture OR crop; 2Grassland OR prairie; 3Alpine OR mountain; 4Wetland OR marsh OR wetlands OR marshes; 5Seawater OR sea OR marine OR ocean; 6Aquifer OR groundwater OR karst OR cave; 7Volcanic OR volcano; 8Atmosphere OR atmospheric; 9Hydrothermal OR geothermal; 10Halophilic OR salt OR saline; 11Psychrophilic OR ice OR glacier OR glacial OR arctic OR permafrost; 12Symbiont OR symbiotic; 13Endophyte OR endophytic; 14Rhizosphere OR root OR rhizospheric; 15Phyllosphere OR phyllospheric OR leaf OR leaves; 16Insect OR larvae OR moth; 17Cow OR bovine OR cattle OR calf; 18Rumen OR ruminal; 19Intestinal OR intestine OR gastrointestinal; 20Mouth OR oral OR buccal; 21Feces OR faeces OR fecal; 22Antibiotic OR antibiotics; 23Degrading OR degradation OR degradative; 24Polluted OR pollution; 25Activated sludge; 26Acid OR acidic OR acidophilic; 27Alkaline OR alkaliphilic; 28Anaerobic OR anaerobe OR anaerobes. The first row of data shows the total number of occurrences of the nucleotide database featuring each phylum or class name in the [Organism] field, with the exclusion of the genomic projects (NOT genome). The taxonomical groups of Armatimonadetes, Caldiserica, and Lentisphaerae, currently in the process of becoming novel phyla, were at this stage dealt with as candidate phyla awaiting placement. [file 2042-5783-1-7-S1.DOC]

|  | Actinobacteria | Aquificae | Bacteroidetes | Chlorobi | Chlamydiae | Verrucomicrobia | Chloroflexi | Cyanobacteria | Deferribacteres | Deinococcus Thermus | Fibrobacter Acidobacteria | Dictyoglomi | Elusimicrobia | Firmicutes | Fusobacteria | Gemmatimonadetes | Nitrospirae | Planctomycetes |
| --- | --- | --- | --- | --- | --- | --- | --- | --- | --- | --- | --- | --- | --- | --- | --- | --- | --- | --- |
| GENBANK | **157719** | **2016** | **50727** | **1951** | **21308** | **3096** | **5395** | **45620** | **156** | **4544** | **13135** | **62** | **334** | **348141** | **3654** | **1646** | **1534** | **7839** |
| Soil | 20870 | 19 | 4894 | 28 | 17 | 1288 | 698 | 1137 | 9 | 95 | 10216 | 8 | 13 | 12316 | 4 | 1362 | 213 | 1806 |
| Agriculture^1^ | 6022 | 31 | 3471 | 19 | 22 | 607 | 188 | 564 | 2 | 34 | 2804 | 13 | 1 | 17894 | 17 | 323 | 105 | 1076 |
| Pasture | 129 | 0 | 135 | 0 | 0 | 132 | 7 | 4 | 0 | 0 | 568 | 0 | 0 | 615 | 0 | 49 | 22 | 136 |
| Grassland^2^ | 780 | 9 | 392 | 0 | 0 | 177 | 10 | 15 | 1 | 0 | 367 | 6 | 1 | 1161 | 0 | 56 | 18 | 157 |
| Forest | 3536 | 6 | 655 | 1 | 1 | 226 | 28 | 133 | 0 | 1 | 1234 | 0 | 0 | 1192 | 0 | 638 | 26 | 311 |
| Alpine^3^ | 1612 | 5 | 1333 | 7 | 103 | 229 | 212 | 864 | 0 | 22 | 1286 | 8 | 4 | 1356 | 0 | 129 | 92 | 199 |
| Wetland^4^ | 1072 | 0 | 1711 | 33 | 1 | 35 | 113 | 452 | 1 | 1 | 379 | 0 | 0 | 1068 | 34 | 52 | 17 | 428 |
| River | 856 | 0 | 707 | 4 | 28 | 76 | 77 | 698 | 0 | 3 | 40 | 0 | 2 | 839 | 1 | 2 | 20 | 305 |
| Lake | 4461 | 1 | 5154 | 225 | 4 | 398 | 103 | 4508 | 3 | 10 | 353 | 0 | 0 | 2902 | 5 | 613 | 50 | 506 |
| Freshwater | 2169 | 0 | 1133 | 17 | 23 | 185 | 100 | 2593 | 3 | 6 | 87 | 2 | 3 | 440 | 8 | 17 | 36 | 841 |
| Seawater^5^ | 7469 | 223 | 7731 | 215 | 40 | 548 | 1152 | 12837 | 48 | 50 | 2741 | 4 | 0 | 6993 | 38 | 59 | 219 | 2672 |
| Sediment | 2548 | 33 | 1773 | 132 | 18 | 246 | 588 | 701 | 16 | 22 | 808 | 4 | 7 | 3186 | 24 | 62 | 127 | 1385 |
| Aquifer^6^ | 803 | 3 | 431 | 4 | 0 | 39 | 144 | 43 | 5 | 4 | 187 | 0 | 4 | 929 | 2 | 15 | 58 | 83 |
| Volcanic^7^ | 157 | 45 | 64 | 0 | 0 | 18 | 12 | 68 | 0 | 4 | 23 | 0 | 0 | 229 | 7 | 4 | 8 | 16 |
| Mine-Ores | 620 | 7 | 94 | 18 | 3 | 38 | 119 | 19 | 6 | 8 | 129 | 0 | 1 | 575 | 0 | 11 | 92 | 50 |
| Desert | 467 | 12 | 172 | 3 | 0 | 13 | 23 | 816 | 0 | 43 | 102 | 0 | 0 | 548 | 0 | 19 | 1 | 12 |
| Arid | 326 | 0 | 104 | 0 | 0 | 3 | 6 | 171 | 0 | 1 | 12 | 0 | 0 | 179 | 0 | 0 | 0 | 1 |
| Atmosphere^8^ | 1792 | 0 | 144 | 0 | 0 | 43 | 5 | 237 | 0 | 1 | 162 | 0 | 0 | 416 | 3 | 42 | 17 | 50 |
| Hydrotherm.^9^ | 87 | 593 | 148 | 16 | 0 | 31 | 100 | 299 | 30 | 65 | 73 | 1 | 1 | 856 | 3 | 4 | 24 | 90 |
| Halophilic^10^ | 924 | 0 | 690 | 63 | 7 | 23 | 154 | 388 | 0 | 11 | 18 | 0 | 0 | 2374 | 0 | 16 | 18 | 83 |
| Thermoph. | 291 | 57 | 100 | 2 | 0 | 1 | 56 | 996 | 7 | 1324 | 2 | 5 | 0 | 2898 | 0 | 6 | 15 | 6 |
| Psychroph.^11^ | 1558 | 0 | 949 | 8 | 2 | 63 | 130 | 451 | 0 | 16 | 246 | 0 | 0 | 861 | 2 | 74 | 24 | 21 |
| Symbiont^12^ | 390 | 0 | 415 | 35 | 3 | 18 | 47 | 788 | 0 | 1 | 2 | 0 | 17 | 974 | 0 | 0 | 2 | 1 |
| Plants | 2661 | 25 | 390 | 96 | 97 | 31 | 72 | 1667 | 0 | 92 | 151 | 0 | 2 | 3464 | 39 | 2 | 30 | 124 |
| Endophyte^13^ | 1208 | 0 | 68 | 0 | 0 | 2 | 0 | 0 | 0 | 1 | 3 | 0 | 0 | 1153 | 0 | 0 | 0 | 0 |
| Rhizosph.^14^ | 3158 | 1 | 675 | 12 | 1 | 159 | 30 | 72 | 5 | 7 | 484 | 0 | 2 | 2419 | 2 | 63 | 20 | 77 |
| Phyllosph.^15^ | 513 | 0 | 45 | 0 | 0 | 2 | 2 | 72 | 0 | 4 | 7 | 0 | 0 | 558 | 0 | 0 | 0 | 0 |
| Insect^16^ | 110 | 0 | 398 | 0 | 2 | 0 | 0 | 7 | 1 | 5 | 0 | 0 | 2 | 1014 | 0 | 0 | 0 | 1 |
| Cow^17^ | 432 | 0 | 1915 | 0 | 23 | 11 | 0 | 10 | 0 | 1 | 3 | 0 | 9 | 1924 | 26 | 0 | 0 | 0 |
| Rumen^18^ | 15 | 0 | 273 | 0 | 0 | 1 | 0 | 0 | 0 | 0 | 47 | 0 | 9 | 883 | 14 | 0 | 0 | 0 |
| Food | 1279 | 1 | 433 | 0 | 14 | 25 | 3 | 108 | 3 | 65 | 4 | 0 | 0 | 16038 | 12 | 0 | 9 | 28 |
| Human | 16167 | 0 | 10272 | 1 | 277 | 6 | 4 | 77 | 11 | 25 | 5 | 0 | 0 | 50797 | 1970 | 2 | 0 | 2 |
| Intestinal^19^ | 1263 | 0 | 3758 | 0 | 6 | 62 | 14 | 6 | 9 | 3 | 4 | 0 | 1 | 9694 | 230 | 1 | 0 | 60 |
| Mouth^20^ | 3022 | 3 | 3514 | 0 | 2 | 0 | 4 | 8 | 11 | 14 | 5 | 0 | 0 | 25949 | 0 | 0 | 0 | 6 |
| Feces^21^ | 1582 | 0 | 7173 | 0 | 57 | 10 | 0 | 0 | 0 | 0 | 2 | 0 | 0 | 5946 | 20 | 0 | 0 | 3 |
| Clinical | 2521 | 0 | 703 | 0 | 497 | 0 | 0 | 0 | 0 | 2 | 2 | 0 | 0 | 6936 | 32 | 0 | 0 | 0 |
| Antibiotic^22^ | 2114 | 1 | 283 | 1 | 8 | 0 | 1 | 2 | 2 | 65 | 2 | 0 | 0 | 1663 | 7 | 0 | 0 | 0 |
| Resistant | 1348 | 5 | 140 | 0 | 4 | 0 | 1 | 24 | 0 | 109 | 2 | 2 | 0 | 7979 | 0 | 0 | 5 | 0 |
| Degrading^23^ | 2449 | 0 | 536 | 4 | 4 | 6 | 44 | 48 | 1 | 4 | 29 | 1 | 0 | 2170 | 15 | 5 | 5 | 14 |
| Heavy metal | 141 | 0 | 69 | 1 | 0 | 3 | 19 | 3 | 0 | 0 | 41 | 0 | 1 | 369 | 0 | 0 | 9 | 10 |
| Polluted^24^ | 393 | 0 | 723 | 1 | 0 | 14 | 11 | 40 | 0 | 3 | 40 | 0 | 1 | 455 | 0 | 8 | 4 | 29 |
| Industrial | 1053 | 12 | 197 | 1 | 5 | 5 | 93 | 55 | 2 | 14 | 79 | 0 | 1 | 3630 | 1 | 2 | 5 | 14 |
| Act. sludge^25^ | 242 | 0 | 364 | 3 | 1 | 11 | 58 | 2 | 0 | 2 | 27 | 0 | 0 | 232 | 0 | 6 | 76 | 46 |
| Acid ^26^ | 3297 | 85 | 368 | 24 | 269 | 144 | 195 | 296 | 3 | 154 | 383 | 0 | 3 | 29045 | 32 | 50 | 175 | 87 |
| Alkaline^27^ | 353 | 0 | 134 | 0 | 0 | 15 | 1 | 54 | 0 | 12 | 1 | 0 | 0 | 1238 | 0 | 0 | 0 | 52 |
| Oxidizing | 126 | 30 | 60 | 21 | 2 | 29 | 8 | 6 | 0 | 5 | 12 | 0 | 0 | 367 | 0 | 11 | 175 | 933 |
| Reducing | 183 | 5 | 115 | 22 | 1 | 3 | 33 | 14 | 15 | 1 | 26 | 1 | 0 | 1075 | 13 | 0 | 39 | 11 |
| Anaerobic^28^ | 401 | 1 | 587 | 18 | 0 | 27 | 158 | 6 | 19 | 4 | 29 | 5 | 0 | 3285 | 43 | 3 | 23 | 1326 |

|  | Alphaproteobacteria | Betaproteobacteria | Gammaproteobacteria | Deltaproteobacteria | Epsilonproteobacteria | Zetaproteobacteria | Spirochaetes | Synergistetes | Tenericutes | Thermodesulfobacteria | Thermotogae | **SUM** | Archaea | SUM Bacteria / Archaea | uncultured | uncultured /  SUM Bacteria |
| --- | --- | --- | --- | --- | --- | --- | --- | --- | --- | --- | --- | --- | --- | --- | --- | --- |
| **GENBANK** | **118172** | **112659** | **329912** | **38272** | **37047** | **10** | **20188** | **487** | **11458** | **145** | **1642** | **1338869** | **180418** | **7.42** | **2143037** | **1.60** |
| Soil | 13799 | 12466 | 14375 | 4391 | 135 | 0 | 41 | 1 | 85 | 2 | 6 | **100294** | 26834 | 3.74 | 466950 | 4.66 |
| Agriculture^1^ | 9953 | 5696 | 10923 | 1398 | 600 | 0 | 208 | 1 | 670 | 2 | 8 | **62652** | 7808 | 8.02 | 80018 | 1.28 |
| Pasture | 441 | 305 | 137 | 88 | 0 | 0 | 0 | 0 | 0 | 0 |  | **2768** | 203 | 13.64 | 5310 | 1.92 |
| Grassland^2^ | 837 | 1226 | 732 | 149 | 9 | 0 | 3 | 0 | 4 | 0 | 2 | **6112** | 1642 | 3.72 | 28885 | 4.73 |
| Forest | 2263 | 1772 | 1213 | 230 | 1 | 0 | 30 | 0 | 39 | 0 | 2 | **13538** | 1110 | 12.20 | 50966 | 3.76 |
| Alpine^3^ | 1503 | 1179 | 1198 | 278 | 16 | 0 | 524 | 0 | 3 | 3 | 0 | **12165** | 850 | 14.31 | 20352 | 1.67 |
| Wetland^4^ | 1219 | 1325 | 2563 | 1086 | 85 | 0 | 26 | 0 | 12 | 0 | 0 | **11713** | 1830 | 6.40 | 28370 | 2.42 |
| River | 830 | 1261 | 2145 | 386 | 62 | 0 | 28 | 0 | 3 | 0 | 0 | **8373** | 3589 | 2.33 | 75203 | 8.98 |
| Lake | 3244 | 4717 | 3693 | 844 | 113 | 0 | 50 | 0 | 3 | 5 | 3 | **31968** | 6635 | 4.82 | 81363 | 2.55 |
| Freshwater | 1241 | 2399 | 1137 | 476 | 37 | 0 | 32 | 0 | 13 | 3 | 0 | **13001** | 2152 | 6.04 | 24130 | 1.86 |
| Seawater^5^ | 13210 | 4132 | 38067 | 4216 | 1499 | 6 | 379 | 28 | 192 | 21 | 67 | **104856** | 26187 | 4.00 | 257662 | 2.46 |
| Sediment | 2363 | 2699 | 6433 | 3510 | 322 | 0 | 114 | 2 | 3 | 5 | 5 | **27136** | 14253 | 1.90 | 89042 | 3.28 |
| Aquifer^6^ | 541 | 921 | 1309 | 1727 | 244 | 0 | 17 | 1 | 0 | 0 | 2 | **7516** | 1314 | 5.72 | 27270 | 3.63 |
| Volcanic^7^ | 216 | 221 | 481 | 92 | 147 | 0 | 1 | 0 | 0 | 0 | 0 | **1813** | 2448 | 0.74 | 11407 | 6.29 |
| Mine-Ores | 404 | 631 | 861 | 184 | 6 | 0 | 27 | 0 | 12 | 0 | 0 | **3915** | 786 | 4.98 | 15168 | 3.87 |
| Desert | 354 | 156 | 228 | 15 | 22 | 0 | 0 | 0 | 3 | 0 | 0 | **3009** | 504 | 5.97 | 8048 | 2.67 |
| Arid | 233 | 90 | 154 | 3 | 0 | 0 | 0 | 0 | 1 | 0 | 0 | **1284** | 189 | 6.79 | 2355 | 1.83 |
| Atmosphere^8^ | 454 | 190 | 183 | 55 | 11 | 0 | 0 | 0 | 0 | 0 | 0 | **3805** | 3072 | 1.24 | 15538 | 4.08 |
| Hydrotherm.^9^ | 210 | 256 | 1268 | 183 | 718 | 4 | 11 | 2 | 9 | 29 | 45 | **5156** | 5788 | 0.89 | 23555 | 4.57 |
| Halophilic^10^ | 1346 | 712 | 3817 | 1234 | 53 | 0 | 101 | 9 | 13 | 0 | 3 | **12057** | 4065 | 2.97 | 15037 | 1.25 |
| Thermoph. | 108 | 149 | 184 | 51 | 198 | 0 | 6 | 27 | 1 | 8 | 237 | **6735** | 1253 | 5.38 | 6232 | 0.93 |
| Psychroph.^11^ | 998 | 1569 | 2968 | 256 | 65 | 0 | 17 | 0 | 7 | 0 | 0 | **10285** | 2866 | 3.59 | 36242 | 3.52 |
| Symbiont^12^ | 3052 | 141 | 2327 | 76 | 23 | 0 | 145 | 2 | 56 | 0 | 0 | **8515** | 129 | 66.01 | 11332 | 1.33 |
| Plants | 3745 | 2757 | 8739 | 515 | 159 | 1 | 99 | 0 | 144 | 0 | 91 | **25193** | 2770 | 9.09 | 36539 | 1.45 |
| Endophyte^13^ | 715 | 261 | 1349 | 1 | 0 | 0 | 0 | 0 | 2 | 0 | 0 | **4763** | 0 | - | 17015 | 3.57 |
| Rhizosph.^14^ | 4839 | 2666 | 3711 | 281 | 6 | 0 | 7 | 9 | 12 | 0 | 1 | **18719** | 3477 | 5.38 | 55726 | 2.98 |
| Phyllosph.^15^ | 667 | 158 | 1409 | 0 | 0 | 0 | 0 | 0 | 353 | 0 | 0 | **3790** | 0 | - | 36120 | 9.53 |
| Insect^16^ | 512 | 49 | 1500 | 7 | 0 | 0 | 52 | 0 | 70 | 0 | 0 | **3730** | 3 | 1243.3 | 3803 | 1.02 |
| Cow^17^ | 573 | 242 | 1547 | 8 | 232 | 0 | 221 | 1 | 141 | 0 | 0 | **7319** | 3329 | 2.20 | 54504 | 7.45 |
| Rumen^18^ | 0 | 62 | 114 | 30 | 3 | 0 | 80 | 4 | 0 | 0 | 0 | **1535** | 3880 | 0.40 | 22886 | 14.91 |
| Food | 1122 | 667 | 9201 | 170 | 853 | 0 | 79 | 1 | 414 | 0 | 3 | **30532** | 2040 | 14.97 | 27610 | 0.90 |
| Human | 2844 | 3750 | 6575 | 88 | 1182 | 0 | 1624 | 255 | 194 | 0 | 0 | **96128** | 1056 | 91.03 | 158817 | 1.65 |
| Intestinal^19^ | 1018 | 1107 | 1950 | 101 | 168 | 0 | 296 | 0 | 31 | 0 | 0 | **19782** | 1120 | 17.66 | 116987 | 5.91 |
| Mouth^20^ | 361 | 1708 | 2698 | 77 | 796 | 0 | 728 | 252 | 37 | 0 | 1 | **39196** | 157 | 249.66 | 4037 | 0.10 |
| Feces^21^ | 27 | 114 | 4087 | 8 | 370 | 0 | 22 | 12 | 36 | 0 | 0 | **19469** | 452 | 43.07 | 474138 | 24.35 |
| Clinical | 1313 | 1520 | 8533 | 7 | 854 | 0 | 269 | 15 | 541 | 0 | 0 | **23745** | 9 | 2638.3 | 3276 | 0.14 |
| Antibiotic^22^ | 427 | 241 | 2172 | 21 | 72 | 0 | 6 | 0 | 10 | 0 | 1 | **7099** | 76 | 93.41 | 10991 | 1.55 |
| Resistant | 496 | 625 | 2964 | 16 | 246 | 0 | 31 | 0 | 112 | 0 | 3 | **14112** | 93 | 151.74 | 33375 | 2.37 |
| Degrading^23^ | 2647 | 2015 | 4512 | 270 | 44 | 0 | 13 | 10 | 10 | 0 | 21 | **14877** | 1274 | 11.68 | 17631 | 1.19 |
| Heavy metal | 181 | 130 | 267 | 77 | 3 | 0 | 58 | 0 | 0 | 0 | 0 | **1382** | 50 | 27.64 | 2147 | 1.55 |
| Polluted^24^ | 503 | 351 | 1013 | 205 | 18 | 0 | 3 | 2 | 1 | 0 | 1 | **3819** | 308 | 12.40 | 7416 | 1.94 |
| Industrial | 644 | 574 | 3309 | 110 | 167 | 0 | 14 | 2 | 73 | 0 | 10 | **10072** | 773 | 13.03 | 28630 | 2.84 |
| Act. sludge^25^ | 727 | 1269 | 872 | 40 | 1 | 0 | 4 | 0 | 0 | 0 | 0 | **3983** | 186 | 21.41 | 30905 | 7.76 |
| Acid ^26^ | 2301 | 2074 | 7191 | 428 | 384 | 1 | 251 | 10 | 119 | 0 | 32 | **47401** | 2346 | 20.21 | 21646 | 0.46 |
| Alkaline^27^ | 386 | 78 | 761 | 64 | 3 | 0 | 26 | 0 | 2 | 0 | 4 | **3184** | 633 | 5.03 | 4663 | 1.46 |
| Oxidizing | 412 | 7827 | 1190 | 170 | 53 | 2 | 2 | 0 | 1 | 0 | 2 | **11444** | 10101 | 1.13 | 27571 | 2.41 |
| Reducing | 201 | 357 | 609 | 4931 | 67 | 0 | 11 | 8 | 1 | 18 | 4 | **7764** | 926 | 8.38 | 16413 | 2.11 |
| Anaerobic^28^ | 255 | 511 | 876 | 679 | 104 | 0 | 68 | 35 | 2 | 1 | 39 | **8505** | 6190 | 1.37 | 308521 | 36.28 |
