# Supplementary material for: Where the bugs are: analyzing distributions of bacterial phyla by descriptor keyword search in the nucleotide database
Source: Microb Inform Exp. 2011 Jul 26;1:7. doi: 10.1186/2042-5783-1-7 (PMC3372287; doi:10.1186/2042-5783-1-7)
Supplement: Additional file 2 — Table S2. Distribution differences in ranks. Elaboration of the data shown in TableS1, expressing the positive (in black) or negative (in red) differences of the percent values with respect to those occurring in the whole database (GenBank column). Upper values: difference over the reference percentage; lower values: fold of increase or decrease of the reference percentage. [file 2042-5783-1-7-S2.DOC]

|  | **GENBANK** | **Soil** | **Agricult.** | **Pasture** | **Grassland** | **Forest** | **Alpine** | **Wetland** | **River** | **Lake** | **Freshwater** | **Seawater** | **Sediment** | **Aquifer** | **Volcanic** | **Mine-Ores** |
| --- | --- | --- | --- | --- | --- | --- | --- | --- | --- | --- | --- | --- | --- | --- | --- | --- |
| **Actinobacteria** | **11.78** | **+9.03** | **-2.17** | **-7.12** | **+0.98** | **+14.34** | **+1.47** | **-2.63** | **-1.56** | **+2.17** | **+4.90** | **-4.66** | **-2.39** | **-1.10** | **-3.12** | **+4.06** |
|  |  | **1.77** | **-1.23** | **-2.53** | **1.08** | **2.22** | **1.12** | **-1.29** | **-1.15** | **1.18** | **1.42** | **-1.65** | **-1.25** | **-1.10** | **-1.36** | **1.34** |
| **Aquificae** | **0.15** | **-0.13** | **-0.10** |  | **-0.003** | **-0.11** | **-0.11** |  |  | **-0.15** |  | **+0.06** | **-0.03** | **-0.11** | **+2.33** | **+0.03** |
|  |  | **-7.95** | **-3.04** |  | **-1.02** | **-3.40** | **-3.66** |  |  | **-48.14** |  | **1.41** | **-1.24** | **-3.77** | **16.48** | **1.19** |
| **Bacteroidetes** | **3.79** | **+1.09** | **+1.75** | **+1.09** | **+2.62** | **+1.05** | **+7.17** | **+10.82** | **+4.66** | **+12.33** | **+4.93** | **+3.58** | **+2.74** | **+1.95** | **-0.26** | **-1.39** |
|  |  | **1.29** | **1.46** | **1.29** | **1.69** | **1.28** | **2.89** | **3.86** | **2.23** | **4.26** | **2.30** | **1.95** | **1.72** | **1.51** | **-1.07** | **-1.58** |
| **Chlorobi** | **0.15** | **-0.12** | **-0.12** |  |  | **-0.14** | **-0.09** | **+0.14** | **-0.10** | **+0.56** | **-0.01** | **+0.06** | **+0.34** | **-0.09** |  | **+0.31** |
|  |  | **-5.22** | **-4.81** |  |  | **-19.73** | **-2.53** | **1.93** | **-3.05** | **4.83** | **-1.11** | **1.41** | **3.34** | **-2.74** |  | **3.16** |
| **Chlamydiae** | **1.59** | **-1.57** | **-1.56** |  |  | **-1.58** | **-0.74** | **-1.58** | **-1.26** | **-1.58** | **-1.41** | **-1.55** | **-1.53** |  |  | **-1.51** |
|  |  | **-93.89** | **-45.32** |  |  | **-215.46** | **-1.88** | **-186.41** | **-4.76** | **-127.19** | **-9.00** | **-41.72** | **-23.99** |  |  | **-20.77** |
| **Verrucomicrobia** | **0.23** | **+1.05** | **+0.74** | **+4.54** | **+2.66** | **+1.44** | **+1.65** | **+0.07** | **+0.68** | **+1.01** | **+1.19** | **+0.29** | **+0.68** | **+0.29** | **+0.76** | **+0.74** |
|  |  | **5.55** | **4.19** | **20.62** | **12.52** | **7.22** | **8.14** | **1.29** | **3.93** | **5.38** | **6.15** | **2.26** | **3.92** | **2.24** | **4.29** | **4.20** |
| **Chloroflexi** | **0.40** | **+0.29** | **-0.10** | **-0.15** | **-0.24** | **-0.20** | **+1.34** | **+0.56** | **+0.52** | **-0.08** | **+0.37** | **+0.70** | **+1.76** | **+1.51** | **+0.26** | **+2.64** |
|  |  | **1.73** | **-1.34** | **-1.59** | **-2.46** | **-1.95** | **4.32** | **2.39** | **2.28** | **-1.25** | **1.91** | **2.73** | **5.38** | **4.75** | **1.64** | **7.54** |
| **Cyanobacteria** | **3.41** | **-2.27** | **-2.51** | **-3.26** | **-3.16** | **-2.42** | **+3.69** | **+0.45** | **+4.93** | **+10.69** | **+16.5** | **+8.84** | **-0.82** | **-2.84** | **+0.34** | **-2.92** |
|  |  | **-3.01** | **-3.79** | **-23.58** | **-13.88** | **-3.47** | **2.08** | **1.13** | **2.45** | **4.14** | **5.85** | **3.59** | **-1.32** | **-5.96** | **1.10** | **-7.02** |
| **Deferribacteres** | **0.01** | **-0.003** | **-0.01** |  | **+0.004** |  |  | **-0.003** |  | **-0.002** | **+0.01** | **+0.03** | **+0.05** | **+0.05** |  | **+0.14** |
|  |  | **-1.30** | **-3.65** |  | **1.40** |  |  | **-1.36** |  | **-1.24** | **1.98** | **3.93** | **5.06** | **5.71** |  | **13.15** |
| **Dein.- Thermus** | **0.34** | **-0.24** | **-0.29** |  |  | **-0.33** | **-0.16** | **-0.33** | **-0.30** | **-0.31** | **-0.29** | **-0.29** | **-0.26** | **-0.29** | **-0.12** | **-0.14** |
|  |  | **-3.58** | **-6.25** |  |  | **-45.95** | **-1.88** | **-39.75** | **-9.47** | **-10.85** | **-7.35** | **-7.12** | **-4.19** | **-6.38** | **-1.54** | **-1.66** |
| **Fibr.- Acidobact.** | **0.98** | **+9.21** | **+3.49** | **+19.5** | **+5.02** | **+8.13** | **+9.59** | **+2.25** | **-0.50** | **+0.12** | **-0.31** | **+1.63** | **+2.00** | **+1.51** | **+0.29** | **+2.31** |
|  |  | **10.38** | **4.56** | **20.92** | **6.12** | **9.29** | **10.78** | **3.30** | **-2.05** | **1.13** | **-1.47** | **2.66** | **3.04** | **2.54** | **1.29** | **3.36** |
| **Dictyoglomi** | **0.005** | **+0.003** | **+0.02** |  | **+0.09** |  | **+0.06** |  |  |  | **+0.01** | **-0.001** | **+0.01** |  |  |  |
|  |  | **1.72** | **4.48** |  | **21.20** |  | **14.20** |  |  |  | **3.32** | **-1.21** | **3.18** |  |  |  |
| **Elusimicrobia** | **0.02** | **-0.01** | **-0.02** |  | **-0.01** |  | **+0.01** |  | **-0.001** |  | **-0.002** |  | **+0.001** | **+0.03** |  | **+0.001** |
|  |  | **-1.92** | **-15.63** |  | **-1.52** |  | **1.32** |  | **-1.04** |  | **-1.08** |  | **1.03** | **2.13** |  | **1.02** |
| **Firmicutes** | **26.00** | **-13.72** | **+2.56** | **-3.78** | **-7.01** | **-17.20** | **-14.86** | **-16.88** | **-15.98** | **-16.92** | **-22.62** | **-19.33** | **-14.26** | **-13.64** | **-13.37** | **-11.32** |
|  |  | **-2.12** | **1.10** | **-1.17** | **-1.37** | **-2.95** | **-2.33** | **-2.85** | **-2.59** | **-2.86** | **-7.68** | **-3.90** | **-2.21** | **-2.10** | **-2.06** | **-1.77** |
| **Fusobacteria** | **0.27** | **-0.27** | **-0.25** |  |  |  |  | **+0.02** | **-0.26** | **-0.26** | **-0.21** | **-0.24** | **-0.18** | **-0.25** | **+0.11** |  |
|  |  | **-68.43** | **-10.06** |  |  |  |  | **1.06** | **-22.85** | **-17.45** | **-4.44** | **-7.53** | **-3.09** | **-10.26** | **1.41** |  |
| **Gemmatimonadet.** | **0.12** | **+1.24** | **+0.39** | **+1.65** | **+0.79** | **+4.59** | **+0.94** | **+0.32** | **-0.10** | **+1.79** | **+0.01** | **-0.07** | **+0.11** | **+0.08** | **+0.10** | **+0.16** |
|  |  | **11.05** | **4.19** | **14.40** | **7.45** | **38.33** | **8.63** | **3.61** | **-5.15** | **15.60** | **1.06** | **-2.18** | **1.86** | **1.62** | **1.79** | **2.29** |
| **Nitrospirae** | **0.11** | **+0.10** | **+0.05** | **+0.68** | **+0.18** | **+0.08** | **+0.64** | **+0.03** | **+0.12** | **+0.04** | **+0.16** | **+0.09** | **+0.35** | **+0.66** | **+0.33** | **+2.24** |
|  |  | **1.85** | **1.46** | **6.94** | **2.57** | **1.68** | **6.60** | **1.27** | **2.08** | **1.37** | **2.42** | **1.82** | **4.08** | **6.74** | **3.85** | **20.51** |
| **Planctomycetes** | **0.59** | **+1.22** | **+1.13** | **+4.33** | **+1.98** | **+1.71** | **+1.05** | **+3.07** | **+3.06** | **+1.00** | **+5.88** | **+1.96** | **+4.52** | **+0.52** | **+0.30** | **+0.69** |
|  |  | **3.08** | **2.93** | **8.39** | **4.39** | **3.92** | **2.79** | **6.24** | **6.22** | **2.70** | **11.05** | **4.35** | **8.72** | **1.89** | **1.51** | **2.18** |
| **Alphaproteobact.** | **8.83** | **+4.93** | **+7.06** | **+7.11** | **+4.87** | **+7.89** | **+3.53** | **+1.58** | **+1.09** | **+1.32** | **+0.72** | **+3.77** | **-0.12** | **-1.63** | **+3.09** | **+1.49** |
|  |  | **1.56** | **1.80** | **1.81** | **1.55** | **1.89** | **1.40** | **1.18** | **1.12** | **1.15** | **1.08** | **1.43** | **-1.01** | **-1.23** | **1.35** | **1.17** |
| **Betaproteobact.** | **8.41** | **+4.01** | **+0.68** | **+2.60** | **+11.6** | **+4.67** | **+1.28** | **+2.90** | **+6.65** | **+6.34** | **+10.0** | **-4.47** | **+1.53** | **+3.84** | **+3.78** | **+7.70** |
|  |  | **1.48** | **1.08** | **1.31** | **2.38** | **1.56** | **1.15** | **1.34** | **1.79** | **1.75** | **2.19** | **-2.14** | **1.18** | **1.46** | **1.45** | **1.92** |
| .  **Gammaproteobact.** | **24.64** | **-10.31** | **-7.21** | **-19.69** | **-12.66** | **-15.68** | **-14.79** | **-2.76** | **+0.98** | **-13.09** | **-15.90** | **+11.6** | **-0.93** | **-7.22** | **+1.89** | **-2.65** |
|  |  | **-1.72** | **-1.41** | **-4.98** | **-2.06** | **-2.75** | **-2.50** | **-1.13** | **1.04** | **-2.13** | **-2.82** | **1.47** | **-1.04** | **-1.41** | **1.08** | **-1.12** |
| **Deltaproteobact.** | **2.86** | **+1.52** | **-0.63** | **+0.32** | **-0.42** | **-1.16** | **-0.57** | **+6.41** | **+1.75** | **-0.22** | **+0.80** | **+1.16** | **+10.0** | **+20.1** | **+2.22** | **+1.84** |
|  |  | **1.53** | **-1.28** | **1.11** | **-1.17** | **-1.68** | **-1.25** | **3.24** | **1.61** | **-1.08** | **1.28** | **1.41** | **4.52** | **8.04** | **1.78** | **1.64** |
| **Epsilonproteobact.** | **2.77** | **-2.63** | **-1.81** |  | **-2.62** | **-2.76** | **-2.64** | **-2.04** | **-2.03** | **-2.41** | **-2.48** | **-1.34** | **-1.58** | **+0.48** | **+5.34** | **-2.61** |
|  |  | **-20.56** | **-2.89** |  | **-18.79** | **-374.60** | **-21.04** | **-3.81** | **-3.74** | **-7.83** | **-9.72** | **-1.94** | **-2.33** | **1.17** | **2.93** | **-18.05** |
| **Zetaproteobact.** | **0.001** |  |  |  |  |  |  |  |  |  |  | **+0.005** |  |  |  |  |
|  |  |  |  |  |  |  |  |  |  |  |  | **7.66** |  |  |  |  |
| **Spirochaetes** | **1.51** | **-1.47** | **-1.18** |  | **-1.46** | **-1.29** | **+2.80** | **-1.29** | **-1.17** | **-1.35** | **-1.26** | **-1.15** | **-1.09** | **-1.28** | **-1.45** | **-0.82** |
|  |  | **-36.88** | **-4.54** |  | **-30.72** | **-6.80** | **2.86** | **-6.79** | **-4.51** | **-9.64** | **-6.13** | **-4.17** | **-3.59** | **-6.67** | **-27.34** | **-2.19** |
| **Synergistetes** | **0.04** | **-0.04** | **-0.03** |  |  |  |  |  |  |  |  | **-0.01** | **-0.03** | **-0.02** |  |  |
|  |  | **-36.48** | **-22.79** |  |  |  |  |  |  |  |  | **-1.36** | **-4.94** | **-2.73** |  |  |
| **Tenericutes** | **0.86** | **-0.77** | **+0.21** |  | **-0.79** | **-0.57** | **-0.83** | **-0.75** | **-0.82** | **-0.85** | **-0.76** | **-0.67** | **-0.84** |  |  | **-0.55** |
|  |  | **-10.10** | **1.25** |  | **-13.08** | **-2.97** | **-34.70** | **-8.35** | **-23.89** | **-91.19** | **-8.56** | **-4.67** | **-77.41** |  |  | **-2.79** |
| **Thermodesulfobac.** | **0.01** | **-0.01** | **-0.01** |  |  |  | **+0.01** |  |  | **+0.004** | **+0.01** | **+0.01** | **+0.01** |  |  |  |
|  |  |  | **-3.39** |  |  |  | **2.28** |  |  | **1.44** | **2.13** | **1.85** | **1.70** |  |  |  |
| **Thermotogae** | **0.12** | **-0.12** | **-0.11** |  | **-0.09** | **-0.11** |  |  |  | **-0.11** |  | **-0.06** | **-0.10** | **-0.10** |  |  |
|  |  | **-20.50** | **-9.60** |  | **-3.75** | **-8.30** |  |  |  | **-13.07** |  | **-1.92** | **-6.66** | **-4.61** |  |  |
|  | | | | | | | | | | | | | | | | |
| **Score Coding** | **>2** | | **>5** | | **>10** | | **>20** | | **>30** | | **<-2** | | **<-10** | | **<-20** | |

|  | **Desert** | **Arid** | **Atmosphere** | **Hydrotherm.** | **Halophilic** | **Thermoph.** | **Psychroph.** | **Symbiont** | **Plants** | **Endophyte** | **Rhizosph.** | **Phyllosph.** | **Insect** | **Cow** | **Rumen** | **Food** |
| --- | --- | --- | --- | --- | --- | --- | --- | --- | --- | --- | --- | --- | --- | --- | --- | --- |
| **Actinobacteria** | **+3.74** | **+13.61** | **+35.32** | **-10.09** | **-4.12** | **-7.46** | **+3.37** | **-7.20** | **-1.22** | **+13.58** | **+5.09** | **+1.76** | **-8.83** | **-5.88** | **-10.80** | **-7.59** |
|  | **1.32** | **2.16** | **4.00** | **-6.98** | **-1.54** | **-2.73** | **1.29** | **-2.57** | **-1.12** | **2.15** | **1.43** | **1.15** | **-3.99** | **-2.00** | **-12.05** | **-2.81** |
| **Aquificae** | **+0.25** |  |  | **+11.35** |  | **+0.70** |  |  | **-0.05** |  | **-0.15** |  |  |  |  | **-0.15** |
|  | **2.65** |  |  | **76.38** |  | **5.62** |  |  | **-1.52** |  | **-28.19** |  |  |  |  | **-45.97** |
| **Bacteroidetes** | **+1.93** | **+4.31** | **-0.004** | **-0.92** | **+1.93** | **-2.30** | **+5.44** | **+1.08** | **-2.24** | **-2.36** | **-0.18** | **-2.60** | **+6.88** | **+22.3** | **+14.0** | **-2.37** |
|  | **1.51** | **2.14** | **-1.00** | **-1.32** | **1.51** | **-2.55** | **2.44** | **1.29** | **-2.45** | **-2.65** | **-1.05** | **-3.19** | **2.82** | **6.91** | **4.69** | **-2.67** |
| **Chlorobi** | **-0.05** |  |  | **+0.16** | **+0.38** | **-0.12** | **-0.07** | **+0.27** | **+0.24** |  | **-0.08** |  |  |  |  |  |
|  | **-1.46** |  |  | **2.13** | **3.59** | **-4.91** | **-1.87** | **2.82** | **2.62** |  | **-2.27** |  |  |  |  |  |
| **Chlamydiae** |  |  |  |  | **-1.53** |  | **-1.57** | **-1.56** | **-1.21** |  | **-1.59** |  | **-1.54** | **-1.28** |  | **-1.55** |
|  |  |  |  |  | **-27.41** |  | **-81.84** | **-45.17** | **-4.13** |  | **-297.9** |  | **-29.68** | **-5.06** |  | **-34.71** |
| **Verrucomicrobia** | **+0.20** | **+0.002** | **+0.90** | **+0.37** | **-0.04** | **-0.22** | **+0.38** | **-0.02** | **-0.11** | **-0.19** | **+0.62** | **-0.18** |  | **-0.08** | **-0.17** | **-0.15** |
|  | **1.87** | **1.01** | **4.89** | **2.60** | **-1.21** | **-15.57** | **2.65** | **-1.09** | **-1.88** | **-5.51** | **3.67** | **-4.38** |  | **-1.54** | **-3.55** | **-2.82** |
| **Chloroflexi** | **+0.36** | **+0.06** | **-0.27** | **+1.54** | **+0.87** | **+0.43** | **+0.86** | **+0.15** | **-0.12** |  | **-0.24** | **-0.35** |  |  |  | **-0.39** |
|  | **1.90** | **1.16** | **-3.07** | **4.81** | **3.17** | **2.06** | **3.14** | **1.37** | **-1.41** |  | **-2.51** | **-7.64** |  |  |  | **-41.01** |
| **Cyanobacteria** | **+23.7** | **+9.91** | **+2.82** | **+2.39** | **-0.19** | **+11.38** | **+0.98** | **+5.85** | **+3.21** |  | **-3.02** | **-1.51** | **-3.22** | **-3.27** |  | **-3.05** |
|  | **7.96** | **3.91** | **1.83** | **1.70** | **-1.06** | **4.34** | **1.29** | **2.72** | **1.94** |  | **-8.86** | **-1.79** | **-18.16** | **-24.94** |  | **-9.63** |
| **Deferribacteres** |  |  |  | **+0.57** |  | **+0.09** |  |  |  |  | **+0.02** |  | **+0.02** |  |  | **0.00** |
|  |  |  |  | **49.94** |  | **8.92** |  |  |  |  | **2.29** |  | **2.30** |  |  | **-1.19** |
| **Dein.- Thermus** | **+1.09** | **-0.26** | **-0.31** | **+0.92** | **-0.25** | **+19.32** | **-0.18** | **-0.33** | **+0.03** | **-0.32** | **-0.30** | **-0.23** | **-0.21** | **-0.33** |  | **-0.13** |
|  | **4.21** | **-4.36** | **-12.91** | **3.71** | **-3.72** | **57.92** | **-2.18** | **-28.90** | **1.08** | **-16.17** | **-9.08** | **-3.22** | **-2.53** | **-24.8** |  | **-1.59** |
| **Fibr.- Acidobact.** | **+2.41** | **-0.05** | **+3.28** | **+0.43** | **-0.83** | **-0.95** | **+1.41** | **-0.96** | **-0.38** | **-0.92** | **+1.60** | **-0.80** |  | **-0.94** | **+2.08** | **-0.97** |
|  | **3.46** | **-1.05** | **4.34** | **1.44** | **-6.57** | **-33.04** | **2.44** | **-41.77** | **-1.64** | **-15.58** | **2.64** | **-5.31** |  | **-23.9** | **3.12** | **-74.88** |
| **Dictyoglomi** |  |  |  | **+0.01** |  | **+0.07** |  |  |  |  |  |  |  |  |  |  |
|  |  |  |  | **4.19** |  | **16.03** |  |  |  |  |  |  |  |  |  |  |
| **Elusimicrobia** |  |  |  | **-0.01** |  |  |  | **+0.17** | **-0.02** |  | **-0.01** |  | **+0.03** | **+0.10** | **+0.56** |  |
|  |  |  |  | **-1.29** |  |  |  | **8.00** | **-3.14** |  | **-2.33** |  | **2.15** | **4.93** | **23.50** |  |
| **Firmicutes** | **-7.79** | **-12.06** | **-15.07** | **-9.40** | **-6.31** | **+17.03** | **-17.63** | **-14.56** | **-12.25** | **-1.80** | **-13.08** | **-11.28** | **+1.18** | **+0.29** | **+31.5** | **+26.5** |
|  | **-1.43** | **-1.87** | **-2.38** | **-1.57** | **-1.32** | **1.65** | **-3.11** | **-2.27** | **-1.89** | **-1.07** | **-2.01** | **-1.77** | **1.05** | **1.01** | **2.21** | **2.02** |
| **Fusobacteria** |  |  | **-0.19** | **-0.21** |  |  | **-0.25** |  | **-0.12** |  | **-0.26** |  |  | **+0.08** | **+0.64** | **-0.23** |
|  |  |  | **-3.46** | **-4.69** |  |  | **-14.03** |  | **-1.76** |  | **-25.54** |  |  | **1.30** | **3.34** | **-6.94** |
| **Gemmatimonadet.** | **+0.51** |  | **+0.98** | **-0.05** | **+0.01** | **-0.03** | **+0.60** |  | **-0.12** |  | **+0.21** |  |  |  |  |  |
|  | **5.14** |  | **8.98** | **-1.58** | **1.08** | **-1.38** | **5.85** |  | **-15.49** |  | **2.74** |  |  |  |  |  |
| **Nitrospirae** | **-0.08** |  | **+0.33** | **+0.35** | **+0.03** | **+0.11** | **+0.12** | **-0.09** | **+0.005** |  | **-0.01** |  |  |  |  | **-0.09** |
|  | **-3.45** |  | **3.90** | **4.06** | **1.30** | **1.94** | **2.04** | **-4.88** | **1.04** |  | **-1.07** |  |  |  |  | **-3.89** |
| **Planctomycetes** | **-0.19** | **-0.51** | **+0.73** | **+1.16** | **+0.10** | **-0.50** | **-0.38** | **-0.57** | **-0.09** |  | **-0.17** |  | **-0.56** |  |  | **-0.49** |
|  | **-1.47** | **-7.52** | **2.24** | **2.98** | **1.18** | **-6.57** | **-2.87** | **-49.85** | **-1.19** |  | **-1.42** |  | **-21.84** |  |  | **-6.38** |
| **Alphaproteobact.** | **+2.94** | **+9.32** | **+3.11** | **-4.75** | **+2.34** | **-7.22** | **+0.88** | **+27.02** | **+6.04** | **+6.19** | **+17.02** | **+8.77** | **+4.90** | **-1.00** |  | **-5.15** |
|  | **1.33** | **2.06** | **1.35** | **-2.17** | **1.26** | **-5.50** | **1.10** | **4.06** | **1.68** | **1.70** | **2.93** | **1.99** | **1.56** | **-1.13** |  | **-2.40** |
| **Betaproteobact.** | **-3.23** | **-1.41** | **-3.42** | **-3.45** | **-2.51** | **-6.20** | **+6.84** | **-6.76** | **+2.53** | **-2.93** | **+5.83** | **-4.25** | **-7.10** | **-5.11** | **-4.38** | **-6.23** |
|  | **-1.62** | **-1.20** | **-1.69** | **-1.69** | **-1.42** | **-3.80** | **1.81** | **-5.08** | **1.30** | **-1.54** | **1.69** | **-2.02** | **-6.41** | **-2.54** | **-2.08** | **-3.85** |
| .  **Gammaproteobact.** | **-17.06** | **-12.65** | **-19.83** | **-0.05** | **+7.02** | **-21.91** | **+4.22** | **+2.69** | **+10.05** | **+3.68** | **-4.82** | **+12.54** | **+15.57** | **-3.50** | **-17.21** | **+5.49** |
|  | **-3.25** | **-2.05** | **-5.12** | **-1.00** | **1.28** | **-9.02** | **1.17** | **1.11** | **1.41** | **1.15** | **-1.24** | **1.51** | **1.63** | **-1.17** | **-3.32** | **1.22** |
| **Deltaproteobact.** | **-2.36** | **-2.62** | **-1.41** | **0.69** | **7.38** | **-2.10** | **-0.37** | **-1.97** | **-0.81** | **-2.84** | **-1.36** |  | **-2.67** | **-2.75** | **-0.90** | **-2.30** |
|  | **-5.73** | **-12.23** | **-1.98** | **1.24** | **3.58** | **-3.77** | **-1.15** | **-3.20** | **-1.40** | **-136.1** | **-1.90** |  | **-15.23** | **-26.15** | **-1.46** | **-5.13** |
| **Epsilonproteobact.** | **-2.04** |  | **-2.48** | **+11.16** | **-2.33** | **+0.17** | **-2.14** | **-2.50** | **-2.14** |  | **-2.73** |  |  | **+0.40** | **-2.57** | **+0.03** |
|  | **-3.78** |  | **-9.57** | **5.03** | **-6.29** | **1.06** | **-4.38** | **-10.24** | **-4.38** |  | **-86.33** |  |  | **1.15** | **-14.16** | **1.01** |
| **Zetaproteobact.** |  |  |  | **+0.08** |  |  |  |  | **+0.003** |  |  |  |  |  |  |  |
|  |  |  |  | **103.8** |  |  |  |  | **5.31** |  |  |  |  |  |  |  |
| **Spirochaetes** |  |  |  | **-1.29** | **-0.67** | **-1.42** | **-1.34** | **+0.20** | **-1.11** |  | **-1.47** |  | **-0.11** | **+1.51** | **+3.70** | **-1.25** |
|  |  |  |  | **-7.07** | **-1.80** | **-16.93** | **-9.12** | **1.13** | **-3.84** |  | **-40.32** |  | **-1.08** | **2.00** | **3.46** | **-5.83** |
| **Synergistetes** |  |  |  | **+0.00** | **+0.04** | **+0.36** |  | **-0.01** |  |  | **+0.01** |  |  | **-0.02** | **+0.22** | **-0.03** |
|  |  |  |  | **1.07** | **2.05** | **11.02** |  | **-1.55** |  |  | **1.32** |  |  | **-2.66** | **7.16** | **-11.11** |
| **Tenericutes** | **-0.76** | **-0.78** |  | **-0.68** | **-0.75** | **-0.84** | **-0.79** | **-0.20** | **-0.28** | **-0.81** | **-0.79** | **+8.46** | **+1.02** | **+1.07** |  | **+0.50** |
|  | **-8.58** | **-10.99** |  | **-4.90** | **-7.94** | **-57.64** | **-12.57** | **-1.30** | **-1.50** | **-20.38** | **-13.35** | **10.88** | **2.19** | **2.25** |  | **1.58** |
| **Thermodesulfobac.** |  |  |  | **+0.55** |  | **+0.11** |  |  |  |  |  |  |  |  |  |  |
|  |  |  |  | **51.93** |  | **10.97** |  |  |  |  |  |  |  |  |  |  |
| **Thermotogae** |  |  |  | **+0.75** | **-0.10** | **+3.40** |  |  | **+0.24** |  | **-0.12** |  |  |  |  | **-0.11** |
|  |  |  |  | **7.12** | **-4.93** | **28.69** |  |  | **2.95** |  | **-22.96** |  |  |  |  | **-12.48** |

|  | **Human** | **Intestinal** | **Mouth** | **Feces** | **Clinical** | **Antibiotic** | **Resistant** | **Degrading** | **Heavy metal** | **Polluted** | **Industrial** | **Act. Sludge** | **Acid** | **Alkaline** | **Oxidizing** | **Reducing** | **Anaerobic** |
| --- | --- | --- | --- | --- | --- | --- | --- | --- | --- | --- | --- | --- | --- | --- | --- | --- | --- |
| **Actinobact.** | **+5.04** | **-5.40** | **-4.07** | **-3.65** | **-1.16** | **+18.00** | **-2.23** | **+4.68** | **-1.58** | **-1.49** | **-1.33** | **-5.70** | **-4.82** | **-0.69** | **-10.68** | **-9.42** | **-7.07** |
|  | **1.43** | **-1.85** | **-1.53** | **-1.45** | **-1.11** | **2.53** | **-1.23** | **1.40** | **-1.15** | **-1.14** | **-1.13** | **-1.94** | **-1.69** | **-1.06** | **-10.70** | **-5.00** | **-2.50** |
| **Aquificae** |  |  | **-0.14** |  |  | **-0.14** | **-0.12** |  |  |  | **-0.03** |  | **+0.03** |  | **+0.11** | **-0.09** | **-0.14** |
|  |  |  | **-19.67** |  |  | **-10.69** | **-4.25** |  |  |  | **-1.26** |  | **1.19** |  | **1.74** | **-2.34** | **-12.81** |
| **Bacteroidetes** | **+6.90** | **+15.21** | **+5.18** | **+33.0** | **-0.83** | **+0.20** | **-2.80** | **-0.19** | **1.20** | **+15.1** | **-1.83** | **+5.35** | **-3.01** | **+0.42** | **-3.26** | **-2.31** | **+3.11** |
|  | **2.82** | **5.01** | **2.37** | **9.72** | **-1.28** | **1.05** | **-3.82** | **-1.05** | **1.32** | **5.00** | **-1.94** | **2.41** | **-4.88** | **1.11** | **-7.23** | **-2.56** | **1.82** |
| **Chlorobi** | **-0.14** |  |  |  |  | **-0.13** |  | **-0.12** | **-0.07** | **-0.12** | **-0.14** | **-0.07** | **-0.10** |  | **+0.04** | **+0.14** | **+0.07** |
|  | **-140.08** |  |  |  |  | **-10.34** |  | **-5.42** | **-2.01** | **-5.57** | **-14.68** | **-1.93** | **-2.88** |  | **1.26** | **1.94** | **1.45** |
| **Chlamydiae** | **-1.30** | **-1.56** | **-1.59** | **-1.30** | **+0.50** | **-1.48** | **-1.56** | **-1.56** |  |  | **-1.54** | **-1.57** | **-1.02** |  | **-1.57** | **-1.58** |  |
|  | **-5.52** | **-52.47** | **-311.9** | **-5.44** | **1.32** | **-14.12** | **-56.15** | **-59.19** |  |  | **-32.06** | **-63.39** | **-2.80** |  | **-91.07** | **-123.6** |  |
| **Verrucomicr.** | **-0.22** | **+0.08** |  | **-0.18** |  |  |  | **-0.19** | **-0.01** | **+0.14** | **-0.18** | **+0.04** | **+0.07** | **+0.24** | **+0.02** | **-0.19** | **+0.09** |
|  | **-37.05** | **1.36** |  | **-4.50** |  |  |  | **-5.73** | **-1.07** | **1.59** | **-4.66** | **1.19** | **1.31** | **2.04** | **1.10** | **-5.98** | **1.37** |
| **Chloroflexi** | **-0.40** | **-0.33** | **-0.39** |  |  | **-0.39** | **-0.40** | **-0.11** | **+0.97** | **-0.11** | **+0.52** | **+1.05** | **+0.01** | **-0.37** | **-0.33** | **+0.02** | **+1.45** |
|  | **-96.84** | **-5.69** | **-39.49** |  |  | **-28.61** | **-56.86** | **-1.36** | **3.41** | **-1.40** | **2.29** | **3.61** | **1.02** | **-12.83** | **-5.76** | **1.05** | **4.61** |
| .  **Cyanobact.** | **-3.33** | **-3.38** | **-3.39** |  |  | **-3.38** | **-3.24** | **-3.08** | **-3.19** | **-2.36** | **-2.86** | **-3.36** | **-2.78** | **-1.71** | **-3.35** | **-3.23** | **-3.34** |
|  | **-42.54** | **-112.3** | **-166.9** |  |  | **-120.9** | **-20.04** | **-10.56** | **-15.70** | **-3.25** | **-6.24** | **-67.86** | **-5.46** | **-2.01** | **-64.99** | **-18.90** | **-48.30** |
| **Deferribact.** | **-0.0002** | **+0.03** | **+0.02** |  |  | **+0.02** |  | **-0.005** |  |  | **+0.01** |  | **-0.01** |  |  | **+0.18** | **+0.21** |
|  | **-1.02** | **3.90** | **2.41** |  |  | **2.42** |  | **-1.73** |  |  | **1.70** |  | **-1.84** |  |  | **16.58** | **19.17** |
| **Dei-Thermus** | **-0.31** | **-0.32** | **-0.30** |  | **-0.33** | **+0.58** | **+0.43** | **-0.31** |  | **-0.26** | **-0.20** | **-0.29** | **-0.01** | **+0.04** | **-0.30** | **-0.33** | **-0.29** |
|  | **-13.05** | **-22.38** | **-9.50** |  | **-40.29** | **2.70** | **2.28** | **-12.62** |  | **-4.32** | **-2.44** | **-6.76** | **-1.04** | **1.11** | **-7.77** | **-26.35** | **-7.22** |
| .  **Fibr-Acidob.** | **-0.98** | **-0.96** | **-0.97** | **-0.97** | **-0.97** | **-0.95** | **-0.97** | **-0.79** | **+1.99** | **+0.07** | **-0.20** | **-0.30** | **-0.17** | **-0.95** | **-0.88** | **-0.65** | **-0.64** |
|  | **-188.61** | **-48.52** | **-76.91** | **-95.50** | **-116.48** | **-34.82** | **-69.22** | **-5.03** | **3.02** | **1.07** | **-1.25** | **-1.45** | **-1.21** | **-31.24** | **-9.36** | **-2.93** | **-2.88** |
| **Dictyoglomi** |  |  |  |  |  |  | **+0.01** | **+0.002** |  |  |  |  |  |  |  | **+0.01** | **+0.05** |
|  |  |  |  |  |  |  | **3.06** | **1.45** |  |  |  |  |  |  |  | **2.78** | **12.70** |
| **Elusimicrob.** |  | **-0.02** |  |  |  |  |  |  | **+0.05** | **+0.001** | **-0.02** |  | **-0.02** |  |  |  |  |
|  |  | **-4.93** |  |  |  |  |  |  | **2.90** | **1.05** | **-2.51** |  | **-3.94** |  |  |  |  |
| **Firmicutes** | **+26.84** | **+23.00** | **+40.20** | **+4.54** | **+3.21** | **-2.58** | **+30.5** | **-11.42** | **+0.70** | **-14.09** | **+10.0** | **-20.18** | **+35.2** | **+12.8** | **-22.80** | **-12.16** | **+12.6** |
|  | **2.03** | **1.88** | **2.55** | **1.17** | **1.12** | **-1.11** | **2.17** | **-1.78** | **1.03** | **-2.18** | **1.39** | **-4.46** | **2.36** | **1.50** | **-8.11** | **-1.88** | **1.49** |
| **Fusobacteria** | **+1.78** | **+0.89** |  | **-0.17** | **-0.14** | **-0.17** |  | **-0.17** |  |  | **-0.26** |  | **-0.21** |  |  | **-0.11** | **+0.23** |
|  | **7.51** | **4.26** |  | **-2.66** | **-2.03** | **-2.77** |  | **-2.71** |  |  | **-27.49** |  | **-4.04** |  |  | **-1.63** | **1.85** |
| **Gemmatimon.** | **-0.12** | **-0.12** |  |  |  |  |  | **-0.09** |  | **+0.09** | **-0.10** | **+0.03** | **-0.02** |  | **-0.03** |  | **-0.09** |
|  | **-59.09** | **-24.32** |  |  |  |  |  | **-3.66** |  | **1.70** | **-6.19** | **1.23** | **-1.17** |  | **-1.28** |  | **-3.49** |
| **Nitrospirae** |  |  |  |  |  |  | **-0.08** | **-0.08** | **+0.54** | **-0.01** | **-0.06** | **+1.79** | **+0.25** |  | **+1.41** | **+0.39** | **+0.16** |
|  |  |  |  |  |  |  | **-3.23** | **-3.41** | **5.68** | **-1.09** | **-2.31** | **16.65** | **3.22** |  | **13.35** | **4.38** | **2.36** |
| **Planctomyc.** | **-0.58** | **-0.28** | **-0.57** | **-0.57** |  |  |  | **-0.49** | **+0.14** | **+0.17** | **-0.45** | **+0.57** | **-0.40** | **+1.05** | **+7.57** | **-0.44** | **+15.0** |
|  | **-281.4** | **-1.93** | **-38.25** | **-38.00** |  |  |  | **-6.22** | **1.24** | **1.30** | **-4.21** | **1.97** | **-3.19** | **2.79** | **13.92** | **-4.13** | **26.63** |
| **Alphaprot.** | **-5.87** | **-3.68** | **-7.91** | **-8.69** | **-3.30** | **-2.81** | **-5.31** | **+8.97** | **+4.27** | **+4.34** | **-2.43** | **+9.43** | **-3.97** | **3.30** | **-5.23** | **-6.24** | **-5.83** |
|  | **-2.98** | **-1.72** | **-9.58** | **-63.64** | **-1.60** | **-1.47** | **-2.51** | **2.02** | **1.48** | **1.49** | **-1.38** | **2.07** | **-1.82** | **1.37** | **-2.45** | **-3.41** | **-2.94** |
| **Betaprot.** | **-4.51** | **-2.82** | **-4.06** | **-7.83** | **-2.01** | **-5.02** | **-3.99** | **+5.13** | **+0.99** | **+0.78** | **-2.72** | **+23.45** | **-4.04** | **-5.96** | **+59.9** | **-3.82** | **-2.41** |
|  | **-2.16** | **-1.50** | **-1.93** | **-14.37** | **-1.31** | **-2.48** | **-1.90** | **1.61** | **1.12** | **1.09** | **-1.48** | **3.79** | **-1.92** | **-3.43** | **8.13** | **-1.83** | **-1.40** |
| .  **Gammaprot.** | **-17.80** | **-14.78** | **-17.76** | **-3.65** | **+11.29** | **+5.95** | **-3.64** | **+5.69** | **-5.32** | **1.88** | **+8.21** | **-2.75** | **-9.47** | **-0.74** | **-14.24** | **-16.80** | **-14.34** |
|  | **-3.60** | **-2.50** | **-3.58** | **-1.17** | **1.46** | **1.24** | **-1.17** | **1.23** | **-1.28** | **1.08** | **1.33** | **-1.13** | **-1.62** | **-1.03** | **-2.37** | **-3.14** | **-2.39** |
| **Deltaprot.** | **-2.77** | **-2.35** | **-2.66** | **-2.82** | **-2.83** | **-2.56** | **-2.75** | **-1.04** | **+2.71** | **+2.51** | **-1.77** | **-1.85** | **-1.96** | **-0.85** | **-1.37** | **+60.65** | **+5.13** |
|  | **-31.23** | **-5.60** | **-14.55** | **-69.57** | **-96.97** | **-9.66** | **-25.21** | **-1.58** | **1.95** | **1.88** | **-2.62** | **-2.85** | **-3.17** | **-1.42** | **-1.92** | **22.22** | **2.79** |
| **Epsilonprot.** | **-1.54** | **-1.92** | **-0.74** | **-0.87** | **+0.83** | **-1.75** | **-1.02** | **-2.47** | **-2.55** | **-2.30** | **-1.11** | **-2.74** | **-1.96** | **-2.67** | **-2.30** | **-1.90** | **-1.54** |
|  | **-2.25** | **-3.26** | **-1.36** | **-1.46** | **1.30** | **-2.73** | **-1.59** | **-9.36** | **-12.75** | **-5.87** | **-1.67** | **-110.2** | **-3.42** | **-29.37** | **-5.97** | **-3.21** | **-2.26** |
| **Zetaprot.** |  |  |  |  |  |  |  |  |  |  |  |  | **+0.001** |  | **+0.02** |  |  |
|  |  |  |  |  |  |  |  |  |  |  |  |  | **2.82** |  | **23.40** |  |  |
| **Spirochaetes** | **+0.18** | **-0.01** | **+0.35** | **-1.39** | **-0.37** | **-1.42** | **-1.29** | **-1.42** | **+2.69** | **-1.43** | **-1.37** | **-1.41** | **-0.98** | **-0.69** | **-1.49** | **-1.37** | **-0.71** |
|  | **1.12** | **-1.01** | **1.23** | **-13.34** | **-1.33** | **-17.84** | **-6.86** | **-17.26** | **2.78** | **-19.19** | **-10.85** | **-15.01** | **-2.85** | **-1.85** | **-86.28** | **-10.64** | **-1.89** |
| **Synergistetes** | **+0.23** |  | **+0.61** | **+0.03** | **+0.03** |  |  | **+0.03** |  | **+0.02** | **-0.02** |  | **-0.02** |  |  | **+0.07** | **+0.38** |
|  | **7.29** |  | **17.68** | **1.69** | **1.74** |  |  | **1.85** |  | **1.44** | **-1.83** |  | **-1.72** |  |  | **2.83** | **11.31** |
| **Tenericutes** | **-0.65** | **-0.70** | **-0.76** | **-0.67** | **+1.42** | **-0.71** | **-0.06** | **-0.79** |  | **-0.83** | **-0.13** |  | **-0.60** | **-0.79** | **-0.85** | **-0.84** | **-0.83** |
|  | **-4.24** | **-5.46** | **-9.07** | **-4.63** | **2.66** | **-6.08** | **-1.08** | **-12.73** |  | **-32.68** | **-1.18** |  | **-3.41** | **-13.62** | **-97.94** | **-66.44** | **-36.39** |
| **Thermodes.** |  |  |  |  |  |  |  |  |  |  |  |  |  |  |  | **0.22** | **0.001** |
|  |  |  |  |  |  |  |  |  |  |  |  |  |  |  |  | **21.41** | **1.09** |
| **Thermotogae** |  |  | **-0.12** |  |  | **-0.11** | **-0.10** | **+0.02** |  | **-0.10** | **-0.02** |  | **-0.06** | **+0.003** | **-0.11** | **-0.07** | **+0.34** |
|  |  |  | **-48.07** |  |  | **-8.71** | **-5.77** | **1.15** |  | **-4.68** | **-1.24** |  | **-1.82** | **1.02** | **-7.02** | **-2.38** | **3.74** |
