# Supplementary material for: Where the bugs are: analyzing distributions of bacterial phyla by descriptor keyword search in the nucleotide database
Source: Microb Inform Exp. 2011 Jul 26;1:7. doi: 10.1186/2042-5783-1-7 (PMC3372287; doi:10.1186/2042-5783-1-7)
Supplement: Additional file 3 — Table S3. Distribution differences in habitats. Elaboration of the data shown in Table S1, expressing, for each of the phyla, the percent of the total GenBank occurrences associated with each given descriptor, and ordered in decreasing abundance. [file 2042-5783-1-7-S3.DOC]

| **Actinobacteria** | | **Forest** | **0.30** | **Plants** | **4.92** | **Seawater** | **17.70** | **Reducing** | **0.61** | **Antibiotic** | **0.004** |
| --- | --- | --- | --- | --- | --- | --- | --- | --- | --- | --- | --- |
| **Soil** | **13.23** | **Reducing** | **0.25** | **Halophilic** | **3.23** | **Lake** | **12.86** | **Rhizosph.** | **0.56** | **Deferribacteres** | |
| **Human** | **10.25** | **Alpine** | **0.25** | **Symbiont** | **1.79** | **Sediment** | **7.95** | **Forest** | **0.52** | **Seawater** | **30.77** |
| **Seawater** | **4.74** | **Resistant** | **0.25** | **Wetland** | **1.69** | **Alpine** | **7.40** | **Desert** | **0.43** | **Hydrotherm.** | **19.23** |
| **Agricult.** | **3.82** | **Aquifer-Cave** | **0.15** | **Soil** | **1.44** | **Forest** | **7.30** | **Heavy metal** | **0.35** | **Anaerobic** | **12.18** |
| **Lake** | **2.83** | **Mouth** | **0.15** | **Acid** | **1.23** | **Freshwater** | **5.98** | **Intestinal** | **0.26** | **Sediment** | **10.26** |
| **Forest** | **2.24** | **Anaerobic** | **0.05** | **Reducing** | **1.13** | **Grassland** | **5.72** | **Volcanic** | **0.22** | **Reducing** | **9.62** |
| **Acid** | **2.09** | **Lake** | **0.05** | **Oxidizing** | **1.08** | **Rhizosph.** | **5.14** | **Polluted** | **0.20** | **Human** | **7.05** |
| **Rhizosph.** | **2.00** | **Rhizosph.** | **0.05** | **Agricult.** | **0.97** | **Acid** | **4.65** | **Grassland** | **0.19** | **Mouth** | **7.05** |
| **Mouth** | **1.92** | **Food** | **0.05** | **Anaerobic** | **0.92** | **Pasture** | **4.26** | **Oxidizing** | **0.15** | **Soil** | **5.77** |
| **Plants** | **1.69** | **Antibiotic** | **0.05** | **Mine-Ores** | **0.92** | **River** | **2.45** | **Pasture** | **0.13** | **Intestinal** | **5.77** |
| **Sediment** | **1.62** | **Bacteroidetes** | | **Freshwater** | **0.87** | **Psychroph.** | **2.03** | **Arid** | **0.11** | **Thermoph.** | **4.49** |
| **Clinical** | **1.60** | **Human** | **20.25** | **Hydrotherm.** | **0.82** | **Intestinal** | **2.00** | **Atmosphere** | **0.09** | **Mine-Ores** | **3.85** |
| **Degrading** | **1.55** | **Seawater** | **15.24** | **Rhizosph.** | **0.62** | **Atmosphere** | **1.39** | **Human** | **0.07** | **Aquifer-Cave** | **3.21** |
| **Freshwater** | **1.38** | **Feces** | **14.14** | **Psychroph.** | **0.41** | **Aquifer-Cave** | **1.26** | **Mouth** | **0.07** | **Rhizosph.** | **3.21** |
| **Antibiotic** | **1.34** | **Lake** | **10.16** | **Alpine** | **0.36** | **Mine-Ores** | **1.23** | **Food** | **0.06** | **Acid** | **1.92** |
| **Atmosphere** | **1.14** | **Soil** | **9.65** | **River** | **0.21** | **Wetland** | **1.13** | **Phyllosph.** | **0.04** | **Freshwater** | **1.92** |
| **Alpine** | **1.02** | **Intestinal** | **7.41** | **Aquifer-Cave** | **0.21** | **Hydrotherm.** | **1.00** | **Alkaline** | **0.02** | **Lake** | **1.92** |
| **Feces** | **1.00** | **Mouth** | **6.93** | **Degrading** | **0.21** | **Plants** | **1.00** | **Resistant** | **0.02** | **Food** | **1.92** |
| **Psychroph.** | **0.99** | **Agricult.** | **6.84** | **Desert** | **0.15** | **Oxidizing** | **0.94** | **Antibiotic** | **0.02** | **Agricult.** | **1.28** |
| **Resistant** | **0.85** | **Cow-Bovine** | **3.78** | **Actd. sludge** | **0.15** | **Anaerobic** | **0.87** | **Cyanobacteria** | | **Industrial** | **1.28** |
| **Food** | **0.81** | **Sediment** | **3.50** | **Thermoph.** | **0.10** | **Food** | **0.81** | **Seawater** | **28.14** | **Antibiotic** | **1.28** |
| **Intestinal** | **0.80** | **Wetland** | **3.37** | **Human** | **0.05** | **Halophilic** | **0.74** | **Lake** | **9.88** | **Wetland** | **0.64** |
| **Endophyte** | **0.77** | **Alpine** | **2.63** | **Industrial** | **0.05** | **Volcanic** | **0.58** | **Freshwater** | **5.68** | **Insect** | **0.64** |
| **Wetland** | **0.68** | **Freshwater** | **2.23** | **Antibiotic** | **0.05** | **Symbiont** | **0.58** | **Plants** | **3.65** | **Degrading** | **0.64** |
| **Industrial** | **0.67** | **Psychroph.** | **1.87** | **Heavy metal** | **0.05** | **Alkaline** | **0.48** | **Soil** | **2.49** | **Grassland** | **0.64** |
| **Halophilic** | **0.59** | **Polluted** | **1.43** | **Polluted** | **0.05** | **Polluted** | **0.45** | **Thermoph.** | **2.18** | **Deinoc.- Thermos** | |
| **River** | **0.54** | **River** | **1.39** | **Forest** | **0.05** | **Desert** | **0.42** | **Alpine** | **1.89** | **Thermoph.** | **29.14** |
| **Aquif.-Cave** | **0.51** | **Clinical** | **1.39** | **Chlamydiae** | | **Cow-Bovine** | **0.36** | **Desert** | **1.79** | **Acid** | **3.39** |
| **Grassland** | **0.49** | **Halophilic** | **1.36** | **Clinical** | **2.33** | **Actd. sludge** | **0.36** | **Symbiont** | **1.73** | **Resistant** | **2.40** |
| **Mine-Ores** | **0.39** | **Rhizosph.** | **1.33** | **Human** | **1.30** | **Feces** | **0.32** | **Sediment** | **1.54** | **Soil** | **2.09** |
| **Phyllosph.** | **0.33** | **Forest** | **1.29** | **Acid** | **1.26** | **Human** | **0.19** | **River** | **1.53** | **Plants** | **2.02** |
| **Desert** | **0.30** | **Anaerobic** | **1.16** | **Alpine** | **0.48** | **Degrading** | **0.19** | **Agricult.** | **1.24** | **Hydrotherm.** | **1.43** |
| **Cow-Bovine** | **0.27** | **Degrading** | **1.06** | **Plants** | **0.46** | **Industrial** | **0.16** | **Wetland** | **0.99** | **Food** | **1.43** |
| **Anaerobic** | **0.25** | **Food** | **0.85** | **Feces** | **0.27** | **Reducing** | **0.10** | **Psychroph.** | **0.99** | **Antibiotic** | **1.43** |
| **Polluted** | **0.25** | **Aquifer-Cave** | **0.85** | **Seawater** | **0.19** | **Heavy metal** | **0.10** | **Halophilic** | **0.85** | **Seawater** | **1.10** |
| **Symbiont** | **0.25** | **Symbiont** | **0.82** | **River** | **0.13** | **Arid** | **0.10** | **Hydrotherm.** | **0.66** | **Desert** | **0.95** |
| **Alkaline** | **0.22** | **Insect** | **0.78** | **Freshwater** | **0.11** | **Phyllosph.** | **0.06** | **Acid** | **0.65** | **Agricult.** | **0.75** |
| **Arid** | **0.21** | **Grassland** | **0.77** | **Cow-Bovine** | **0.11** | **Endophyte** | **0.06** | **Atmosphere** | **0.52** | **Human** | **0.55** |
| **Thermoph.** | **0.18** | **Plants** | **0.77** | **Agricult.** | **0.10** | **Thermoph.** | **0.03** | **Arid** | **0.37** | **Alpine** | **0.48** |
| **Actd. sludge** | **0.15** | **Acid** | **0.73** | **Sediment** | **0.08** | **Rumen** | **0.03** | **Forest** | **0.29** | **Sediment** | **0.48** |
| **Reducing** | **0.12** | **Actd. sludge** | **0.72** | **Soil** | **0.08** | **Chloroflexi** | | **Food** | **0.24** | **Psychroph.** | **0.35** |
| **Volcanic** | **0.10** | **Antibiotic** | **0.56** | **Food** | **0.07** | **Seawater** | **21.35** | **Human** | **0.17** | **Mouth** | **0.31** |
| **Heavy metal** | **0.09** | **Rumen** | **0.54** | **Antibiotic** | **0.04** | **Soil** | **12.94** | **Rhizosph.** | **0.16** | **Industrial** | **0.31** |
| **Pasture** | **0.08** | **Industrial** | **0.39** | **Halophilic** | **0.03** | **Sediment** | **10.90** | **Phyllosph.** | **0.16** | **Alkaline** | **0.26** |
| **Oxidizing** | **0.08** | **Desert** | **0.34** | **Intestinal** | **0.03** | **Alpine** | **3.93** | **Volcanic** | **0.15** | **Halophilic** | **0.24** |
| **Insect** | **0.07** | **Hydrotherm.** | **0.29** | **Industrial** | **0.02** | **Acid** | **3.61** | **Industrial** | **0.12** | **Lake** | **0.22** |
| **Hydrotherm.** | **0.06** | **Atmosphere** | **0.28** | **Lake** | **0.02** | **Agricult.** | **3.48** | **Alkaline** | **0.12** | **Mine-Ores** | **0.18** |
| **Rumen** | **0.01** | **Resistant** | **0.28** | **Resistant** | **0.02** | **Anaerobic** | **2.93** | **Degrading** | **0.11** | **Rhizosph.** | **0.15** |
| **Aquificae** | | **Pasture** | **0.27** | **Degrading** | **0.02** | **Halophilic** | **2.85** | **Aquifer-Cave** | **0.09** | **Freshwater** | **0.13** |
| **Hydrotherm.** | **29.41** | **Alkaline** | **0.26** | **Symbiont** | **0.01** | **Aquifer-Cave** | **2.67** | **Polluted** | **0.09** | **Oxidizing** | **0.11** |
| **Seawater** | **11.06** | **Reducing** | **0.23** | **Mine-Ores** | **0.01** | **Psychroph.** | **2.41** | **Resistant** | **0.05** | **Insect** | **0.11** |
| **Acid** | **4.22** | **Arid** | **0.21** | **Oxidizing** | **0.01** | **Mine-Ores** | **2.21** | **Mine-Ores** | **0.04** | **Anaerobic** | **0.09** |
| **Thermoph.** | **2.83** | **Thermoph.** | **0.20** | **Psychroph.** | **0.01** | **Wetland** | **2.09** | **Grassland** | **0.03** | **Volcanic** | **0.09** |
| **Volcanic** | **2.23** | **Mine-Ores** | **0.19** | **Insect** | **0.01** | **Lake** | **1.91** | **Reducing** | **0.03** | **Aquifer-Cave** | **0.09** |
| **Sediment** | **1.64** | **Heavy metal** | **0.14** | **Mouth** | **0.01** | **Hydrotherm.** | **1.85** | **Cow-Bovine** | **0.02** | **Phyllosph.** | **0.09** |
| **Agricult.** | **1.54** | **Endophyte** | **0.13** | **Reducing** | **0.004** | **Freshwater** | **1.85** | **Mouth** | **0.02** | **Degrading** | **0.09** |
| **Oxidizing** | **1.49** | **Volcanic** | **0.13** | **Wetland** | **0.004** | **Industrial** | **1.72** | **Insect** | **0.02** | **River** | **0.07** |
| **Plants** | **1.24** | **Oxidizing** | **0.12** | **Actd. sludge** | **0.004** | **River** | **1.43** | **Oxidizing** | **0.01** | **Intestinal** | **0.07** |
| **Soil** | **0.94** | **Phyllosph.** | **0.09** | **Rhizosph.** | **0.004** | **Plants** | **1.33** | **Anaerobic** | **0.01** | **Polluted** | **0.07** |
| **Desert** | **0.60** | **Chlorobi** | | **Forest** | **0.004** | **Actd. sludge** | **1.08** | **Intestinal** | **0.01** | **Actd. sludge** | **0.04** |
| **Industrial** | **0.60** | **Lake** | **11.53** | **Verrucomicrobia** | | **Thermoph.** | **1.04** | **Pasture** | **0.01** | **Clinical** | **0.04** |
| **Grassland** | **0.45** | **Seawater** | **11.02** | **Soil** | **41.60** | **Symbiont** | **0.87** | **Heavy metal** | **0.01** | **Reducing** | **0.02** |
| **Mine-Ores** | **0.35** | **Sediment** | **6.77** | **Agricult.** | **19.61** | **Degrading** | **0.82** | **Actd. sludge** | **0.00** | **Atmosphere** | **0.02** |

| **Wetland** | **0.02** | **Resistant** | **3.23** | **Mine-Ores** | **0.17** | **Degrading** | **0.30** | **Aquifer-Cave** | **1.06** | **Arid** | **0.20** |
| --- | --- | --- | --- | --- | --- | --- | --- | --- | --- | --- | --- |
| **Symbiont** | **0.02** | **Hydrotherm.** | **1.61** | **Phyllosph.** | **0.16** | **Hydrotherm.** | **0.24** | **Rhizosph.** | **0.98** | **Volcanic** | **0.18** |
| **Cow-Bovine** | **0.02** | **Reducing** | **1.61** | **Desert** | **0.16** | **Volcanic** | **0.24** | **Intestinal** | **0.77** | **Hydrotherm.** | **0.18** |
| **Endophyte** | **0.02** | **Degrading** | **1.61** | **Polluted** | **0.13** | **Anaerobic** | **0.18** | **Alkaline** | **0.66** | **Reducing** | **0.17** |
| **Arid** | **0.02** | **Elusimiobia** | | **Freshwater** | **0.13** | **River** | **0.12** | **Atmosphere** | **0.64** | **Heavy metal** | **0.15** |
| **Forest** | **0.02** | **Symbiont** | **5.09** | **Atmosphere** | **0.12** | **Human** | **0.12** | **Mine-Ores** | **0.64** | **Thermoph.** | **0.09** |
| **Fibr.- Acidobacteria** | | **Soil** | **3.89** | **Heavy metal** | **0.11** | **Plants** | **0.12** | **Actd. sludge** | **0.59** | **Feces** | **0.02** |
| **Soil** | **77.78** | **Cow-Bovine** | **2.69** | **Oxidizing** | **0.11** | **Industrial** | **0.12** | **Polluted** | **0.37** | **Betaproteobact.** | |
| **Agricult.** | **21.35** | **Rumen** | **2.69** | **Actd. sludge** | **0.07** | **Intestinal** | **0.06** | **Food** | **0.36** | **Soil** | **11.07** |
| **Seawater** | **20.87** | **Sediment** | **2.10** | **Volcanic** | **0.07** | **Nitrospirae** | | **Psychroph.** | **0.27** | **Oxidizing** | **6.95** |
| **Alpine** | **9.79** | **Alpine** | **1.20** | **Arid** | **0.05** | **Seawater** | **14.28** | **Volcanic** | **0.20** | **Agricult.** | **5.06** |
| **Forest** | **9.39** | **Aquifer-Cave** | **1.20** | **Fusobteria** | | **Soil** | **13.89** | **Industrial** | **0.18** | **Lake** | **4.19** |
| **Sediment** | **6.15** | **Acid** | **0.90** | **Human** | **53.91** | **Acid** | **11.41** | **Degrading** | **0.18** | **Seawater** | **3.67** |
| **Pasture** | **4.32** | **Freshwater** | **0.90** | **Intestinal** | **6.29** | **Oxidizing** | **11.41** | **Desert** | **0.15** | **Human** | **3.33** |
| **Rhizosph.** | **3.68** | **River** | **0.60** | **Anaerobic** | **1.18** | **Sediment** | **8.28** | **Reducing** | **0.14** | **Plants** | **2.45** |
| **Acid** | **2.92** | **Insect** | **0.60** | **Plants** | **1.07** | **Agricult.** | **6.84** | **Heavy metal** | **0.13** | **Sediment** | **2.40** |
| **Wetland** | **2.89** | **Plants** | **0.60** | **Seawater** | **1.04** | **Alpine** | **6.00** | **Thermoph.** | **0.08** | **Rhizosph.** | **2.37** |
| **Grassland** | **2.79** | **Rhizosph.** | **0.60** | **Wetland** | **0.93** | **Mine-Ores** | **6.00** | **Mouth** | **0.08** | **Freshwater** | **2.13** |
| **Lake** | **2.69** | **Agricult.** | **0.30** | **Acid** | **0.88** | **Actd. sludge** | **4.95** | **Feces** | **0.04** | **Acid** | **1.84** |
| **Psychroph.** | **1.87** | **Hydrotherm.** | **0.30** | **Clinical** | **0.88** | **Aquifer-Cave** | **3.78** | **Human** | **0.03** | **Degrading** | **1.79** |
| **Aquifer-Cave** | **1.42** | **Intestinal** | **0.30** | **Cow-Bovine** | **0.71** | **Lake** | **3.26** | **Symbiont** | **0.01** | **Forest** | **1.57** |
| **Atmosphere** | **1.23** | **Industrial** | **0.30** | **Sediment** | **0.66** | **Reducing** | **2.54** | **Insect** | **0.01** | **Mouth** | **1.52** |
| **Plants** | **1.15** | **Heavy metal** | **0.30** | **Feces** | **0.55** | **Freshwater** | **2.35** | **Arid** | **0.01** | **Psychroph.** | **1.39** |
| **Mine-Ores** | **0.98** | **Polluted** | **0.30** | **Agricult.** | **0.47** | **Plants** | **1.96** | **Alphaproteobact.** | | **Clinical** | **1.35** |
| **Desert** | **0.78** | **Grassland** | **0.30** | **Degrading** | **0.41** | **Forest** | **1.69** | **Soil** | **11.68** | **Wetland** | **1.18** |
| **Freshwater** | **0.66** | **Mine-Ores** | **0.30** | **Rumen** | **0.38** | **Hydrotherm.** | **1.56** | **Seawater** | **11.18** | **Actd. sludge** | **1.13** |
| **Industrial** | **0.60** | **Firmicutes** | | **Reducing** | **0.36** | **Psychroph.** | **1.56** | **Agricult.** | **8.42** | **River** | **1.12** |
| **Hydrotherm.** | **0.56** | **Human** | **14.59** | **Food** | **0.33** | **Anaerobic** | **1.50** | **Rhizosph.** | **4.09** | **Grassland** | **1.09** |
| **Rumen** | **0.36** | **Acid** | **8.34** | **Freshwater** | **0.22** | **Pasture** | **1.43** | **Plants** | **3.17** | **Alpine** | **1.05** |
| **Heavy metal** | **0.31** | **Mouth** | **7.45** | **Volcanic** | **0.19** | **River** | **1.30** | **Lake** | **2.75** | **Intestinal** | **0.98** |
| **River** | **0.30** | **Agricult.** | **5.14** | **Antibiotic** | **0.19** | **Rhizosph.** | **1.30** | **Symbiont** | **2.58** | **Aquif.-Cave** | **0.82** |
| **Polluted** | **0.30** | **Food** | **4.61** | **Lake** | **0.14** | **Halophilic** | **1.17** | **Human** | **2.41** | **Halophilic** | **0.63** |
| **Anaerobic** | **0.22** | **Soil** | **3.54** | **Soil** | **0.11** | **Grassland** | **1.17** | **Degrading** | **2.24** | **Food** | **0.59** |
| **Degrading** | **0.22** | **Intestinal** | **2.78** | **Hydrotherm.** | **0.08** | **Atmosphere** | **1.11** | **Sediment** | **2.00** | **Mine-Ores** | **0.56** |
| **Actd. sludge** | **0.21** | **Resistant** | **2.29** | **Atmosphere** | **0.08** | **Wetland** | **1.11** | **Acid** | **1.95** | **Resistant** | **0.55** |
| **Reducing** | **0.20** | **Seawater** | **2.01** | **Psychroph.** | **0.05** | **Thermoph.** | **0.98** | **Forest** | **1.92** | **Industrial** | **0.51** |
| **Volcanic** | **0.18** | **Clinical** | **1.99** | **Aquif.-Cave** | **0.05** | **Food** | **0.59** | **Alpine** | **1.27** | **Anaerobic** | **0.45** |
| **Halophilic** | **0.14** | **Feces** | **1.71** | **Rhizosph.** | **0.05** | **Heavy metal** | **0.59** | **Halophilic** | **1.14** | **Reducing** | **0.32** |
| **Oxidizing** | **0.09** | **Industrial** | **1.04** | **River** | **0.03** | **Volcanic** | **0.52** | **Clinical** | **1.11** | **Polluted** | **0.31** |
| **Arid** | **0.09** | **Plants** | **0.99** | **Industrial** | **0.03** | **Industrial** | **0.33** | **Freshwater** | **1.05** | **Pasture** | **0.27** |
| **Phyllosph.** | **0.05** | **Anaerobic** | **0.94** | **Gemmatimonadet.** | | **Resistant** | **0.33** | **Wetland** | **1.03** | **Endophyte** | **0.23** |
| **Human** | **0.04** | **Sediment** | **0.92** | **Soil** | **82.75** | **Degrading** | **0.33** | **Food** | **0.95** | **Hydrotherm.** | **0.23** |
| **Mouth** | **0.04** | **Lake** | **0.83** | **Forest** | **38.76** | **Polluted** | **0.26** | **Intestinal** | **0.86** | **Cow-Bovine** | **0.21** |
| **Intestinal** | **0.03** | **Thermoph.** | **0.83** | **Lake** | **37.24** | **Symbiont** | **0.13** | **Psychroph.** | **0.84** | **Antibiotic** | **0.21** |
| **Food** | **0.03** | **Rhizosph.** | **0.69** | **Agricult.** | **19.62** | **Desert** | **0.07** | **Grassland** | **0.71** | **Volcanic** | **0.20** |
| **Cow-Bovine** | **0.02** | **Halophilic** | **0.68** | **Alpine** | **7.84** | **Planctomycetes** | | **River** | **0.70** | **Atmosphere** | **0.17** |
| **Endophyte** | **0.02** | **Degrading** | **0.62** | **Psychroph.** | **4.50** | **Seawater** | **34.09** | **Actd. sludge** | **0.62** | **Phyllosph.** | **0.14** |
| **Thermoph.** | **0.02** | **Cow-Bovine** | **0.55** | **Rhizosph.** | **3.83** | **Soil** | **23.04** | **Endophyte** | **0.61** | **Desert** | **0.14** |
| **Symbiont** | **0.02** | **Antibiotic** | **0.48** | **Sediment** | **3.77** | **Sediment** | **17.67** | **Phyllosph.** | **0.56** | **Thermoph.** | **0.13** |
| **Feces** | **0.02** | **Alpine** | **0.39** | **Seawater** | **3.58** | **Anaerobic** | **16.92** | **Industrial** | **0.54** | **Symbiont** | **0.13** |
| **Resistant** | **0.02** | **Alkaline** | **0.36** | **Grassland** | **3.40** | **Agricult.** | **13.73** | **Cow-Bovine** | **0.48** | **Heavy metal** | **0.12** |
| **Antibiotic** | **0.02** | **Forest** | **0.34** | **Wetland** | **3.16** | **Oxidizing** | **11.90** | **Aquifer-Cave** | **0.46** | **Feces** | **0.10** |
| **Clinical** | **0.02** | **Grassland** | **0.33** | **Acid** | **3.04** | **Freshwater** | **10.73** | **Insect** | **0.43** | **Arid** | **0.08** |
| **Alkaline** | **0.01** | **Endophyte** | **0.33** | **Pasture** | **2.98** | **Lake** | **6.45** | **Polluted** | **0.43** | **Alkaline** | **0.07** |
| **Dictyoglomi** | | **Reducing** | **0.31** | **Atmosphere** | **2.55** | **Wetland** | **5.46** | **Resistant** | **0.42** | **Rumen** | **0.06** |
| **Agricult.** | **20.97** | **Wetland** | **0.31** | **Desert** | **1.15** | **Forest** | **3.97** | **Atmosphere** | **0.38** | **Insect** | **0.04** |
| **Soil** | **12.90** | **Insect** | **0.29** | **Freshwater** | **1.03** | **River** | **3.89** | **Pasture** | **0.37** | **Gammaproteobac.** | |
| **Alpine** | **12.90** | **Symbiont** | **0.28** | **Halophilic** | **0.97** | **Alpine** | **2.54** | **Antibiotic** | **0.36** | **Seawater** | **11.54** |
| **Grassland** | **9.68** | **Aquifer-Cave** | **0.27** | **Aquif.-Cave** | **0.91** | **Grassland** | **2.00** | **Oxidizing** | **0.35** | **Soil** | **4.36** |
| **Anaerobic** | **8.06** | **Rumen** | **0.25** | **Oxidizing** | **0.67** | **Pasture** | **1.73** | **Mine-Ores** | **0.34** | **Agricult.** | **3.31** |
| **Thermoph.** | **8.06** | **Psychroph.** | **0.25** | **Mine-Ores** | **0.67** | **Plants** | **1.58** | **Alkaline** | **0.33** | **Food** | **2.79** |
| **Seawater** | **6.45** | **Hydrotherm.** | **0.25** | **Polluted** | **0.49** | **Hydrotherm.** | **1.15** | **Mouth** | **0.31** | **Plants** | **2.65** |
| **Sediment** | **6.45** | **River** | **0.24** | **Thermoph.** | **0.36** | **Acid** | **1.11** | **Desert** | **0.30** | **Clinical** | **2.59** |
| **Freshwater** | **3.23** | **Pasture** | **0.18** | **Actd. sludge** | **0.36** | **Halophilic** | **1.06** | **Anaerobic** | **0.22** | **Acid** | **2.18** |

| **Human** | **1.99** | **Oxidizing** | **0.44** | **Alkaline** | **0.01** | **Halophilic** | **1.85** | **Freshwater** | **2.07** | **Desert** | **0.28** |
| --- | --- | --- | --- | --- | --- | --- | --- | --- | --- | --- | --- |
| **Sediment** | **1.95** | **Food** | **0.44** | **Rumen** | **0.01** | **Rhizosph.** | **1.85** | **Soil** | **1.38** | **Feces** | **0.25** |
| **Degrading** | **1.37** | **Grassland** | **0.39** | **Heavy metal** | **0.01** | **Reducing** | **1.64** | **Agricult.** | **1.38** | **Polluted** | **0.17** |
| **Feces** | **1.24** | **Industrial** | **0.29** | **Actd. sludge** | **0.002** | **Rumen** | **0.82** | **Anaerobic** | **0.69** | **Pasture** | **0.11** |
| **Halophilic** | **1.16** | **Intestinal** | **0.26** | **Forest** | **0.002** | **Hydrotherm.** | **0.41** | **Thermotogae** | | **Arid** | **0.10** |
| **Rhizosph.** | **1.12** | **Volcanic** | **0.24** | **Zetaproteobact.** | | **Sediment** | **0.41** | **Thermoph.** | **14.43** | **Act. sludge** | **0.10** |
| **Lake** | **1.12** | **Human** | **0.23** | **Seawater** | **60.00** | **Symbiont** | **0.41** | **Plants** | **5.54** | **Mouth** | **0.09** |
| **Industrial** | **1.00** | **Pasture** | **0.23** | **Hydrotherm.** | **40.00** | **Industrial** | **0.41** | **Seawater** | **4.08** | **Symbiont** | **0.07** |
| **Psychroph.** | **0.90** | **Mouth** | **0.20** | **Oxidizing** | **20.00** | **Polluted** | **0.41** | **Hydrotherm.** | **2.74** | **Resistant** | **0.05** |
| **Resistant** | **0.90** | **Heavy metal** | **0.20** | **Acid** | **10.00** | **Soil** | **0.21** | **Anaerobic** | **2.38** | **Antibiotic** | **0.04** |
| **Mouth** | **0.82** | **Symbiont** | **0.20** | **Plants** | **10.00** | **Agricult.** | **0.21** | **Acid** | **1.95** | **Heavy metal** | **0.03** |
| **Wetland** | **0.78** | **Alkaline** | **0.17** | **Spirochaetes** | | **Aquifer-Cave** | **0.21** | **Degradino** | **1.28** | **Clinical** | **0.004** |
| **Symbiont** | **0.71** | **Atmosphere** | **0.14** | **Human** | **8.04** | **Cow-Bovine** | **0.21** | **Industrial** | **0.61** | **Insect** | **0.001** |
| **Antibiotic** | **0.66** | **Thermoph.** | **0.13** | **Mouth** | **3.61** | **Food** | **0.21** | **Agricult.** | **0.49** | **Uncultured** | |
| **River** | **0.65** | **Actd. sludge** | **0.10** | **Alpine** | **2.60** | **Tenericutes** | | **Soil** | **0.37** | **Feces** | **22.12** |
| **Intestinal** | **0.59** | **Rumen** | **0.08** | **Seawater** | **1.88** | **Agricult.** | **5.85** | **Sediment** | **0.30** | **Soil** | **21.79** |
| **Cow-Bovine** | **0.47** | **Antibiotic** | **0.05** | **Intestinal** | **1.47** | **Clinical** | **4.72** | **Alcaline** | **0.24** | **Anaerobic** | **14.40** |
| **Insect** | **0.45** | **Resistant** | **0.04** | **Clinical** | **1.33** | **Food** | **3.61** | **Reducing** | **0.24** | **Seawater** | **12.02** |
| **Phyllosph.** | **0.43** | **Desert** | **0.04** | **Acid** | **1.24** | **Phyllosph.** | **3.08** | **Halophilic** | **0.18** | **Human** | **7.41** |
| **Endophyte** | **0.41** | **Feces** | **0.02** | **Cow-Bovine** | **1.09** | **Human** | **1.69** | **Lake** | **0.18** | **Intestinal** | **5.46** |
| **Aquifer-Cave** | **0.40** | **Cow-Bovine** | **0.02** | **Agricult.** | **1.03** | **Seawater** | **1.68** | **Food** | **0.18** | **Sediment** | **4.15** |
| **Hydrotherm.** | **0.38** | **Insect** | **0.02** | **Symbiont** | **0.72** | **Plants** | **1.26** | **Resistano** | **0.18** | **Lake** | **3.80** |
| **Forest** | **0.37** | **Clinical** | **0.02** | **Sediment** | **0.56** | **Cow-Bovine** | **1.23** | **Oxidizing** | **0.12** | **Agricult.** | **3.73** |
| **Alpine** | **0.36** | **Arid** | **0.01** | **Halophilic** | **0.50** | **Acid** | **1.04** | **Aquifer-Cave** | **0.12** | **River** | **3.51** |
| **Oxidizing** | **0.36** | **Endophyte** | **0.002** | **Plants** | **0.49** | **Resistano** | **0.98** | **Grassland** | **0.12** | **Rhizosph.** | **2.60** |
| **Freshwater** | **0.34** | **Epsilonproteobact.** | | **Rumen** | **0.40** | **Soil** | **0.74** | **Forest** | **0.12** | **Cow-Bovine** | **2.54** |
| **Polluted** | **0.31** | **Seawater** | **4.05** | **Food** | **0.39** | **Industrial** | **0.64** | **Mouth** | **0.06** | **Forest** | **2.38** |
| **Anaerobic** | **0.27** | **Human** | **3.19** | **Anaerobic** | **0.34** | **Insect** | **0.61** | **Rhizosph.** | **0.06** | **Plants** | **1.71** |
| **Actd. sludge** | **0.26** | **Clinical** | **2.31** | **Heavy metal** | **0.29** | **Symbiont** | **0.49** | **Antibiotic** | **0.06** | **Psychroph.** | **1.69** |
| **Mine-Ores** | **0.26** | **Food** | **2.30** | **Insect** | **0.26** | **Forest** | **0.34** | **Polluted** | **0.06** | **Phyllosph.** | **1.69** |
| **Alkaline** | **0.23** | **Mouth** | **2.15** | **Lake** | **0.25** | **Mouth** | **0.32** | **Archaea** | | **Resistant** | **1.56** |
| **Grassland** | **0.22** | **Hydrotherm.** | **1.94** | **Soil** | **0.20** | **Feces** | **0.31** | **Soil** | **14.87** | **Act. sludge** | **1.44** |
| **Reducing** | **0.18** | **Agricult.** | **1.62** | **Freshwater** | **0.16** | **Intestinal** | **0.27** | **Seawater** | **14.51** | **Grassland** | **1.35** |
| **Volcanic** | **0.15** | **Acid** | **1.04** | **Resistant** | **0.15** | **Halophilic** | **0.11** | **Sediment** | **7.90** | **Industrial** | **1.34** |
| **Heavy metal** | **0.08** | **Feces** | **1.00** | **Forest** | **0.15** | **Freshwater** | **0.11** | **Oxidizing** | **5.60** | **Wetland** | **1.32** |
| **Desert** | **0.07** | **Sediment** | **0.87** | **River** | **0.14** | **Wetland** | **0.10** | **Agricult.** | **4.33** | **Food** | **1.29** |
| **Thermoph.** | **0.06** | **Resistant** | **0.66** | **Mine-Ores** | **0.13** | **Rhizosph.** | **0.10** | **Lake** | **3.68** | **Oxidizing** | **1.29** |
| **Atmosphere** | **0.06** | **Aquif.-Cave** | **0.66** | **Alkaline** | **0.13** | **Mine-Ores** | **0.10** | **Anaerobic** | **3.43** | **Aquif.-Cave** | **1.27** |
| **Arid** | **0.05** | **Cow-Bovine** | **0.63** | **Wetland** | **0.13** | **Degradino** | **0.09** | **Hydrotherm.** | **3.21** | **Freshwater** | **1.13** |
| **Pasture** | **0.04** | **Thermoph.** | **0.53** | **Feces** | **0.11** | **Antibiotic** | **0.09** | **Halophilic** | **2.25** | **Hydrotherm.** | **1.10** |
| **Rumen** | **0.03** | **Intestinal** | **0.45** | **Psychroph.** | **0.08** | **Hydrotherm.** | **0.08** | **Rumen** | **2.15** | **Rumen** | **1.07** |
| **Deltaproteobact.** | | **Industrial** | **0.45** | **Aquif.-Cave** | **0.08** | **Psychroph.** | **0.06** | **River** | **1.99** | **Acid** | **1.01** |
| **Reducing** | **12.88** | **Plants** | **0.43** | **Industrial** | **0.07** | **Grassland** | **0.03** | **Rhizosph.** | **1.93** | **Alpine** | **0.95** |
| **Soil** | **11.47** | **Volcanic** | **0.40** | **Degrading** | **0.06** | **Desert** | **0.03** | **Cow-Bovine** | **1.85** | **Degrading** | **0.82** |
| **Seawater** | **11.02** | **Soil** | **0.36** | **Hydrotherm.** | **0.05** | **Alpine** | **0.03** | **Atmosphere** | **1.70** | **Endophyte** | **0.79** |
| **Sediment** | **9.17** | **Lake** | **0.31** | **Reducing** | **0.05** | **Sediment** | **0.03** | **Psychroph.** | **1.59** | **Reducing** | **0.77** |
| **Aquifer-Cave** | **4.51** | **Anaerobic** | **0.28** | **Rhizosph.** | **0.03** | **Lake** | **0.03** | **Plants** | **1.54** | **Atmosphere** | **0.73** |
| **Agricult.** | **3.65** | **Wetland** | **0.23** | **Thermoph.** | **0.03** | **River** | **0.03** | **Volcanic** | **1.36** | **Mine-Ores** | **0.71** |
| **Halophilic** | **3.22** | **Antibiotic** | **0.19** | **Antibiotic** | **0.03** | **Alcaline** | **0.02** | **Acid** | **1.30** | **Halophilic** | **0.70** |
| **Wetland** | **2.84** | **Reducing** | **0.18** | **Actd. sludge** | **0.02** | **Anaerobic** | **0.02** | **Freshwater** | **1.19** | **Volcanic** | **0.53** |
| **Lake** | **2.21** | **Psychroph.** | **0.18** | **Polluted** | **0.01** | **Endophyte** | **0.02** | **Food** | **1.13** | **Symbiont** | **0.53** |
| **Anaerobic** | **1.77** | **River** | **0.17** | **Grassland** | **0.01** | **Oxidizing** | **0.01** | **Wetland** | **1.01** | **Antibiotic** | **0.51** |
| **Plants** | **1.35** | **Oxidizing** | **0.14** | **Oxidizing** | **0.01** | **Reducing** | **0.01** | **Grassland** | **0.91** | **Desert** | **0.38** |
| **Freshwater** | **1.24** | **Halophilic** | **0.14** | **Volcanic** | **0.004** | **Thermoph.** | **0.01** | **Aquifer-Cave** | **0.73** | **Polluted** | **0.35** |
| **Acid** | **1.12** | **Degrading** | **0.12** | **Synergistetes** | | **Polluted** | **0.01** | **Degrading** | **0.71** | **Thermoph.** | **0.29** |
| **River** | **1.01** | **Freshwater** | **0.10** | **Human** | **52.36** | **Arid** | **0.01** | **Thermoph.** | **0.69** | **Pasture** | **0.25** |
| **Rhizosph.** | **0.73** | **Symbiont** | **0.06** | **Mouth** | **51.75** | **Thermodesulfobact.** | | **Intestinal** | **0.62** | **Alkaline** | **0.22** |
| **Alpine** | **0.73** | **Desert** | **0.06** | **Anaerobic** | **7.19** | **Hydrotherm.** | **20.00** | **Forest** | **0.62** | **Mouth** | **0.19** |
| **Degrading** | **0.71** | **Polluted** | **0.05** | **Seawater** | **5.75** | **Seawater** | **14.48** | **Human** | **0.59** | **Insect** | **0.18** |
| **Psychroph.** | **0.67** | **Alpine** | **0.04** | **Thermoph.** | **5.54** | **Reducing** | **12.41** | **Reducing** | **0.51** | **Clinical** | **0.15** |
| **Forest** | **0.60** | **Atmosphere** | **0.03** | **Clinical** | **3.08** | **Thermoph.** | **5.52** | **Alpine** | **0.47** | **Arid** | **0.11** |
| **Polluted** | **0.54** | **Grassland** | **0.02** | **Feces** | **2.46** | **Sediment** | **3.45** | **Mine-Ores** | **0.44** | **Heavy metal** | **0.10** |
| **Mine-Ores** | **0.48** | **Rhizosph.** | **0.02** | **Acid** | **2.05** | **Lake** | **3.45** | **Industrial** | **0.43** |  |  |
| **Hydrotherm.** | **0.48** | **Mine-Ores** | **0.02** | **Degrading** | **2.05** | **Alpine** | **2.07** | **Alkaline** | **0.35** |  |  |
