# Supplementary material for: Where the bugs are: analyzing distributions of bacterial phyla by descriptor keyword search in the nucleotide database
Source: Microb Inform Exp. 2011 Jul 26;1:7. doi: 10.1186/2042-5783-1-7 (PMC3372287; doi:10.1186/2042-5783-1-7)
Supplement: Additional file 4 — Table S4. Ecological indexing of the descriptor-generated subcommunities. Elaboration of the data shown in Table S1. Each of the numerical communities individuated by the database filtering with the different descriptors (i.e. each of the rows of Table S1) was treated as a defined ecological assemblage and the following indexes were calculated. Simpson's Inverse Dominance (1/D, Hill's N2); Shannon-Wiener's Diversity (H'), Simpson's Evenness (E1/D); Shannon-Pielou's Evenness (J'). [file 2042-5783-1-7-S4.DOC]

| **Simpson 1/D** | | **Shannon Diversity H'** | | **Simpson Evenness** | | **Shannon Evenness** | |
| --- | --- | --- | --- | --- | --- | --- | --- |
| **10.4809** | **Alpine** | **2.4864** | **Alpine** | **0.6250** | **Endophyte** | **0.8300** | **Alpine** |
| **8.3331** | **Lake** | **2.3847** | **Hydrotherm.** | **0.5240** | **Alpine** | **0.8232** | **Pasture** |
| **8.2470** | **Sediment** | **2.3524** | **Sediment** | **0.5067** | **Pasture** | **0.8017** | **Endophyte** |
| **8.2312** | **Wetland** | **2.2939** | **Wetland** | **0.4902** | **Lake** | **0.7998** | **Lake** |
| **7.7970** | **Soil** | **2.2788** | **Volcanic** | **0.4586** | **Soil** | **0.7941** | **River** |
| **7.6549** | **Hydrotherm.** | **2.2660** | **Lake** | **0.4573** | **River** | **0.7867** | **Forest** |
| **7.5549** | **Freshwater** | **2.2529** | **Mine-Ores** | **0.4471** | **Arid** | **0.7835** | **Soil** |
| **7.4363** | **Volcanic** | **2.2251** | **Aquifer** | **0.4387** | **Forest** | **0.7790** | **Wetland** |
| **7.4242** | **Aquifer** | **2.2199** | **Soil** | **0.4332** | **Wetland** | **0.7739** | **Volcanic** |
| **7.3882** | **Grassland** | **2.2171** | **Freshwater** | **0.4105** | **Grassland** | **0.7610** | **Sediment** |
| **7.3491** | **Mine-Ores** | **2.2016** | **River** | **0.3914** | **Volcanic** | **0.7519** | **Grassland** |
| **7.3174** | **River** | **2.1812** | **Forest** | **0.3860** | **Heavy metal** | **0.7483** | **Heavy metal** |
| **7.0938** | **Pasture** | **2.1732** | **Grassland** | **0.3807** | **Phyllosph.** | **0.7428** | **Aquifer** |
| **7.0193** | **Forest** | **2.1724** | **Pasture** | **0.3777** | **Freshwater** | **0.7426** | **Arid** |
| **6.5620** | **Heavy metal** | **2.1703** | **GENBANK** | **0.3749** | **Sediment** | **0.7408** | **Hydrotherm.** |
| **6.3716** | **Psychroph.** | **2.1202** | **Heavy metal** | **0.3712** | **Aquifer** | **0.7401** | **Freshwater** |
| **6.3576** | **Desert** | **2.1195** | **Psychroph.** | **0.3595** | **Rhizosph.** | **0.7321** | **Agricult.** |
| **6.2893** | **Polluted** | **2.1160** | **Agricult.** | **0.3539** | **Clinical** | **0.7288** | **Mine-Ores** |
| **6.2600** | **Arid** | **2.1143** | **Seawater** | **0.3532** | **Desert** | **0.7281** | **Desert** |
| **6.2264** | **Agricult.** | **2.1044** | **Desert** | **0.3494** | **Polluted** | **0.7198** | **Psychroph.** |
| **6.1999** | **GENBANK** | **2.0403** | **Halophilic** | **0.3459** | **Agricult.** | **0.7073** | **Clinical** |
| **5.7527** | **Rhizosph.** | **2.0354** | **Polluted** | **0.3421** | **Cow** | **0.7058** | **Seawater** |
| **5.6886** | **Halophilic** | **2.0297** | **Anaerobic** | **0.3353** | **Psychroph.** | **0.7042** | **Polluted** |
| **5.4761** | **Seawater** | **2.0030** | **Plants** | **0.3340** | **Mine-Ores** | **0.7005** | **Phyllosph.** |
| **5.2939** | **Plants** | **1.9596** | **Arid** | **0.3153** | **Antibiotic** | **0.6944** | **Rhizosph.** |
| **5.2117** | **Degrading** | **1.9252** | **Rhizosph.** | **0.3066** | **Degrading** | **0.6922** | **GENBANK** |
| **5.1311** | **Cow** | **1.8887** | **Act.d sludge** | **0.3062** | **Hydrotherm.** | **0.6917** | **Cow** |
| **5.0638** | **Act.d sludge** | **1.8732** | **Cow** | **0.2979** | **Act.d sludge** | **0.6702** | **Halophilic** |
| **4.9483** | **Anaerobic** | **1.8475** | **Atmosphere** | **0.2913** | **Symbiont** | **0.6667** | **Anaerobic** |
| **4.5683** | **Phyllosph.** | **1.8252** | **Thermoph.** | **0.2885** | **Insect** | **0.6666** | **Act.d sludge** |
| **4.3749** | **Endophyte** | **1.8174** | **Degrading** | **0.2795** | **Alkaline** | **0.6663** | **Atmosphere** |
| **4.3695** | **Symbiont** | **1.7908** | **Symbiont** | **0.2754** | **Feces** | **0.6613** | **Symbiont** |
| **4.2463** | **Clinical** | **1.7575** | **Clinical** | **0.2738** | **Seawater** | **0.6480** | **Plants** |
| **4.1926** | **Alkaline** | **1.7539** | **Alkaline** | **0.2709** | **Halophilic** | **0.6477** | **Alkaline** |
| **4.0989** | **Antibiotic** | **1.7406** | **Phyllosph.** | **0.2696** | **GENBANK** | **0.6414** | **Degrading** |
| **3.9730** | **Thermoph.** | **1.6854** | **Industrial** | **0.2411** | **Intestinal** | **0.6278** | **Antibiotic** |
| **3.8752** | **Industrial** | **1.6191** | **Intestinal** | **0.2406** | **Plants** | **0.6135** | **Intestinal** |
| **3.8349** | **Atmosphere** | **1.6103** | **Antibiotic** | **0.2397** | **Atmosphere** | **0.6128** | **Insect** |
| **3.7502** | **Insect** | **1.5717** | **Insect** | **0.2356** | **Anaerobic** | **0.5995** | **Thermoph.** |
| **3.5796** | **Feces** | **1.5600** | **Endophyte** | **0.2280** | **Industrial** | **0.5949** | **Industrial** |
| **3.3761** | **Intestinal** | **1.5528** | **Human** | **0.2210** | **Resistant** | **0.5884** | **Human** |
| **3.0643** | **Human** | **1.4515** | **Acid** | **0.2189** | **Human** | **0.5622** | **Rumen** |
| **2.6994** | **Food** | **1.4420** | **Rumen** | **0.2066** | **Rumen** | **0.5584** | **Feces** |
| **2.6860** | **Rumen** | **1.4322** | **Feces** | **0.1975** | **Mouth** | **0.5453** | **Resistant** |
| **2.6517** | **Resistant** | **1.3601** | **Reducing** | **0.1928** | **Food** | **0.5235** | **Mouth** |
| **2.4660** | **Acid** | **1.3551** | **Resistant** | **0.1892** | **Thermoph.** | **0.5134** | **Food** |
| **2.3127** | **Reducing** | **1.3549** | **Food** | **0.1208** | **Oxidizing** | **0.4629** | **Acid** |
| **2.1728** | **Mouth** | **1.2554** | **Mouth** | **0.1101** | **Reducing** | **0.4468** | **Reducing** |
| **2.0539** | **Oxidizing** | **1.2333** | **Oxidizing** | **0.1072** | **Acid** | **0.4353** | **Oxidizing** |
